# How Moral Beliefs Influence Collective Violence. Evidence From Lynching in Mexico

**DOI:** 10.1177/00104140231223747

**Published:** 2023-12-27

**Authors:** Enzo Nussio

**Affiliations:** 31053 ETH Zurich, Zurich, Switzerland

**Keywords:** morality, harm avoidance, collective violence, emotions, survey, Mexico

## Abstract

How do moral beliefs influence favorability to collective violence? In this article, I argue that, first, moral beliefs are influential depending on their salience, as harm avoidance is a common moral concern. The more accessible moral beliefs in decision-making, the more they restrain harmful behavior. Second, moral beliefs are influential depending on their content. Group-oriented moral beliefs can overturn the harm avoidance principle and motivate individuals to favor collective violence. Analysis is based on a representative survey in Mexico City and focuses on a proximate form of collective violence, locally called lynching. Findings support both logics of moral influence. Experimentally induced moral salience reduces favorability to lynching, and group-oriented moral beliefs are related to more favorability. Against existing theories that downplay the relevance of morality and present it as cheap talk, these findings demonstrate how moral beliefs can both restrain and motivate collective violence.

## Introduction

Collective violence is often justified with moral claims. The military invasion of a country, for example, is framed as an act of self-defense, rioters derive their legitimacy from perceived injustice, and mobs attack thieves to provide deserved punishment. Appealing to a greater good, such as protection, justice, and punishment, makes the infliction of harm look necessary (Fiske & Rai, 2014; Ginges, 2019). These examples show that morality plays a key role for collective violence. After all, morality concerns our beliefs about right and wrong behavior, including the acceptable use of violence (Dill & Schubiger, 2021). However, political scientists have rarely studied the effects of moral beliefs on violence. They rather examine morality as an attribute of violence, focusing on the conditions under which violence is perceived to be moral or legitimate – as empirical political scientists have done (Dill & Schubiger, 2021; VandenBerg, 2021), or focusing on the normative conditions under which war is moral or justified – as political theorists have done (Bellamy, 2006; e.g. Lazar, 2017).

In this study, I ask how the moral beliefs individuals hold influence their favorability to collective violence. I thus focus on morality as a driver of violence rather than an attribute of violence. Despite its central importance, this question has received surprisingly little attention. Why? On the one hand, moral beliefs are an intricate aspect of dominant theories on collective violence and are thus hard to separate from other key concepts. Theories focusing on identity (Horowitz, 1985), grievances (Cederman et al., 2013), culture (Huntington, 2011), ideology (Maynard, 2019), and religion (Fox, 2004) subsume morality under a broader category. Identity scholars, for instance, often refer to clashing moral worldviews of rival groups; scholars of religion see religious beliefs as source of morality; and grievance scholars make important assumptions about injustice and group loyalty. However, they do not intend to disentangle moral aspects from these broader categories.

On the other hand, moral beliefs are an exceptionally challenging object for empirical inquiry. Some scholars claim that moral beliefs represent merely post-hoc justification with no explanatory weight for behavior (Swidler, 1986; Walter, 2017). According to this view, expressing moral concerns to justify violent action is cheap talk. Others argue that moral beliefs are a spurious factor. Given that morality is an adaptation to social context, resulting behavior is determined by precisely that context and not morality (Tilly, 2003). Yet another group of scholars views moral beliefs as a consequence of violence rather than a cause of it (Solomon et al., 1991; Staub, 1999). These empirical challenges in part explain why students of collective violence have eschewed moral beliefs.

With the present study, I focus on the moral beliefs held by individuals. They harbor an important explanation for collective violence, as collective violence often faces problems of collective action, given the risks implied in personal participation (Wood, 2003). Moral beliefs are one candidate that can help individuals overcome such barriers. The first contribution of this study is theoretical: I offer a theory of how moral beliefs can influence favorability to collective violence, distinct from theories about identity, grievances, and other dominant theories. This study’s findings are thus relevant for theories on collective violence that embed aspects of morality. The second contribution is empirical: I devise a strategy that overcomes the profound challenges to empirical inquiry, by comparing individuals living under the same contextual conditions and using experimentation to discard post-hoc justification and reverse causation.

Against existing theories that downplay the relevance of morality or subsume it under broader categories, I argue that moral beliefs can influence an individual’s favorability to collective violence through two distinct logics: moral salience and group-oriented moral beliefs. First, moral beliefs are influential if they are a salient ingredient of decision-making. Decision-making draws on different types of beliefs as input (Ajzen, 1991). When moral beliefs are salient, meaning when they are on one’s mind, they should act like a brake on violence, given that harm avoidance is a common – some argue universal – moral concern. Mainstream psychology, in fact, views morality as a “muscle” that restrains harmful behavior (Baumeister, 1999). Bandura (2016) even argues that to inflict harm, a person needs to morally disengage, suggesting that morality can be switched either on or off. Authors focusing on such restraint, a key insight in moral disengagement theory, tend to see the moral domain as limited to universal values of harm avoidance and fairness. In line with this thinking, I argue that moral salience reduces favorability to collective violence. Observing such an empirical pattern would challenge rationalist and materialist theories, which make us believe that moral concerns have no bearing on violence.

Second, and distinct from the logic of moral salience, the content of moral beliefs can also be influential. In contradiction to claims to moral universalism (Kohlberg, 1981), which are baked into the moral salience logic, moral beliefs vary across societies, communities, and individuals, reflecting moral pluralism (Graham et al., 2011). This is an important insight often attributed to anthropologist Richard Shweder et al. (1997), but noted much earlier for example by Edward Westermarck (1908). This variation allows us to examine how certain kinds of moral beliefs influence violence. One of the most universally held moral principles is avoidance of harm, which should reduce favorability to violence, in accordance with the logic of moral salience. However, overriding moral goods, especially if they relate to groups, can motivate individuals to inflict harm (Fiske & Rai, 2014; Hoover et al., 2021). Hence, I argue that group-oriented moral beliefs, stressing collective values such as loyalty and authority more than individual goods, increase favorability to collective violence. If moral concerns were mere cheap talk, as several authors make us believe, such a relationship would not exist.

I further understand individual moral judgement as containing an emotional dimension (Haidt, 2003; Prinz, 2009), which allows for the specification of additional observable implications. To the extent that moral salience *proscribes* collective violence, it should be accompanied by emotions of disapprobation, such as compassion for the targets of violence. Similarly, to the extent that group-oriented moral beliefs *prescribe* collective violence against threats, they should be accompanied by emotional approbation. This may result in decreased levels of compassion and increased anger for the targets of violence.

The implications of the two logics of moral influence are distinct and examining them requires different empirical designs, as I discuss below. However, I integrate both logics as they are, in my view, two fundamental ways through which individually held moral beliefs can influence collective violence and as they draw on two distinct understandings of morality as either universalist or pluralist.

At least two additional logics of moral influence have been studied in the past. First, support for violence may depend on moral conviction and the related moralization of specific issues (Mooijman et al., 2018a; Skitka & Mullen, 2002), including the existence of sacred values (Ginges et al., 2011). Second, support for violence may depend on the type of moral reasoning, i.e. consequentialist versus deontological ethics (Dill et al., 2022; Ginges, 2019). Baron et al. (2022) applied this logic to the case of vigilantism. The last two logics are not analyzed in this study but should be examined further in the future.

To examine expectations in a naturalistic setting, I focus on mob violence in Mexico City, locally called lynching. This type of violence is prevalent across many countries, particularly in imperfect democracies of the global south (Jung & Cohen, 2020). While it is largely absent from news reporting in wealthy Western countries, residents of Indonesia, South Africa, and Mexico repeatedly hear about lynching (Pfeifer, 2017). Different from the more commonly studied historical lynching in the US (Tolnay & Beck, 1995), contemporary lynching in Mexico is not usually defined by the act of killing and most often amounts to a collective beating of a thief by civilians, according to an original event dataset on lynching (Nussio & Clayton, 2022). In the eyes of many residents of Latin America, petty delinquents are representatives of the wider “crime epidemic”, a key threat to society not sufficiently addressed by the state (Godoy, 2006). In such a context, a thief is turned into “everybody’s thief” and thus requires extraordinary countermeasures (Gamallo, 2020, p. 195). More broadly speaking, lynching acts are a “form of political expression for people without access to formal legal venues, a critique of the democratic state and its claim to a rule of law” (Goldstein, 2003, p. 23).

Compared to other forms of collective violence, lynching presents us with two key advantages for the study of morality. First, lynching in Mexico is not usually related to ethnicity or racially motivated as most historical cases in the US (Cook et al., 2018), given that perpetrators and targets tend to be co-ethnics (Zizumbo-Colunga, 2015). Lynching in Mexico thus provides an opportunity to focus on that aspect of informal identity-formation which draws on moral concerns – including group-oriented moral beliefs of loyalty – rather than the commonly studied ascribed identity focusing on ethnicity or religion. Also, while participants often refer to defending their neighborhood and making justice (Godoy, 2006), material incentives and organizational constraints are less relevant. Moral beliefs are thus a likely explanation for lynching.

Second, lynching has been part of Mexico’s landscape of violence for decades (Kloppe-Santamaría, 2020). According to the survey used for this study, roughly 10% of respondents in Mexico City – a considerable number – admit having participated in at least a mild version of collective punishment of a thief, and 71% agree with this practice, likely because the police and justice system are seen as ineffective and corrupt (Azaola Garrido, 2006; D. E. Davis, 2013). Impunity for engagement in lynching may further facilitate this practice, as lynching participants are rarely arrested (Nussio & Clayton, 2022). Hence, I can tap into previously formed opinion and inquire about both support for and participation in lynching. Existing research focuses almost exclusively on support (Dow et al., 2023; Freire & Skarbek, 2023). While the focus on lynching may limit the study’s generalizability, it provides a unique window into the dynamics of morality and collective violence. A comprehensive explanation of the phenomenon of lynching is, though, not a prime objective of this article. For that purpose, readers should consult recently published studies (e.g. Fujii, 2021; Jung & Cohen, 2020; Kloppe-Santamaría, 2020; N. R. Smith, 2019). While not focusing on moral beliefs as causal driver, previous studies have studied the moral legitimacy of lynching. Jung and Cohen (2020), for example, examined the role of state legitimacy for lynching, and Weaver (2019) showed how publicity of lynching gradually undermined the legitimacy of this practice in the Southern US.

A representative survey with 2183 residents of Mexico City provides the main evidence. Given the distinct nature of moral salience and group-oriented moral beliefs, I use different research designs to examine their implications. Moral salience can be tested by temporarily heightening the accessibility of moral beliefs. I thus embed a moral priming experiment in the survey. I find that respondents who are primed for morality are less favorable to lynching and less willing to participate, guarding against concerns about social desirability bias. The moral priming also stimulates compassion for the target of violence, suggesting emotional disapprobation.

Group-oriented moral beliefs are hard to manipulate experimentally as the content of moral beliefs is relatively stable.^1^ I therefore use an observational design to study the second logic of moral influence, dedicating special attention to context-related confounding. Moral beliefs are measured with the tools of moral foundations theory (Graham et al., 2011). Observational analysis shows that respondents who privilege group values (including loyalty, authority, and purity) more than individual values (including harm avoidance and fairness) are more favorable to lynching. The observational approach allows to examine actual participation in lynching, which is also related to group-oriented moral beliefs. Furthermore, group-based morals are associated with less compassion for targets of violence, suggesting emotional approbation of violence.

These findings are largely in line with the two logics of moral influence. Moral beliefs are thus a plausible driver of favorability to collective violence.^2^

## Theory

Before I describe the two logics of moral influence in more detail, I clarify conceptual building blocks. Morality is a universal trait of human societies that has emerged as evolutionary advantage to solve problems of cooperation (Greene, 2013). However, there is large debate about what is moral. Simplifying this debate, scholars have either focused on moral universals, including fairness and harm avoidance (Kohlberg, 1981), or assumed a plural understanding of morality, where right and wrong depend on context (Shweder et al., 1997). The two logics of moral influence presented below draw inspiration from a universalist understanding (moral salience) and a pluralist understanding (group-oriented moral beliefs).

Collective violence is understood as the *intentional infliction of harm on others by a group*. I focus on collective violence, rather than individual acts of violence, as moral beliefs fulfill important functions for social cooperation (Haidt, 2013), and help individuals to overcome barriers to collective action (Olson, 1965; Wood, 2003). Moral beliefs have thus, a priori, potential for facilitating collective action, including collective violence. Collective violence comes in many forms, ranging from aerial bombings to targeted assassinations, from gang rape to social cleansing, executed by military organizations, clandestine networks, and spontaneously convened mobs (Tilly, 2003). My intention is not to explain all forms of collective violence, but to examine whether moral beliefs are a driving force in at least some cases.

### Moral Salience and Collective Violence

Given that “behavior is a function of salient information, or beliefs, relevant to the behavior” (Ajzen, 1991, p. 189), moral beliefs can only be influential if they are a relevant input for decision-making. This is a basic insight from social psychology. While we hold any number of beliefs, those that are accessible, or salient, in the moment of deciding are most influential.

How can moral salience influence individual favorability to collective violence? Violence directly contradicts an almost universally held moral belief, namely that inflicting harm is wrong. One can thus conclude that violence is always immoral, as it contradicts this moral principle (Bandura, 2016; Baumeister, 1999). According to this view, grounded in moral universalism (Kohlberg, 1981), moral barriers inhibit the use of violence. Baumeister and Exline (1999) argue that by internalizing moral rules, individuals develop self-control, or what they call the “moral muscle”. Moral disengagement theory is based on the same rationale, arguing that inflicting harm requires disengagement from moral principles, akin to switching off morality (Bandura, 2016).

Other disciplines have used similar theories to explain violence. Classical criminological theory uses the concept of moral neutralization, which allows criminals to do what they do without being inhibited by moral concerns (Sykes & Matza, 1957). More recent control theory views beliefs in the moral validity of social rules as restraint for crime (Antonaccio & Tittle, 2008; Hirschi, 2002). In some versions of civilization theory, self-control is presented as the psychological foundation of a long-term decline of violence in Western Europe (Eisner, 2014). These broader observations about morality and violence are in line with a proscriptive function of moral beliefs, which tell us what we should *not* do (Janoff-Bulman & Carnes, 2013), namely we should avoid harm or not use violence.

From this perspective, I can derive a testable hypothesis about moral salience. When moral beliefs are accessible, one should be less favorable to the use of collective violence, given that harm avoidance is a key moral concern for most individuals. In contradiction to theories that stress rationalist and materialist drivers of violence and downplay the relevance of moral concerns, I thus propose the following hypothesis:

Hypothesis 1a: Moral salience reduces individual favorability to collective violence.

Favorability has an affective dimension, which is consistent with the sensibility theory of morality. Sensibility theorists state that moral judgments always contain emotions, including feelings of approbation and disapprobation (Prinz, 2009). For example, when we judge actions of others, such as rule transgressions, we can feel emotions like anger or contempt. These emotions can motivate us to either participate in violence against wrongdoers or not (Jasper, 2018). If it is true that moral salience reduces favorability to collective violence, it should come along with emotions of disapprobation for the use of violence.

Emotions have long been related to collective violence. Historical studies about moral outrage, for instance, have been used to explain crowd violence like riots (N. Z. Davis, 1973; Thompson, 1971). Indignation and resentment are described as drivers of rebellion (Costalli & Ruggeri, 2015; Petersen, 2002). Anger and discontent have been associated with theories about relative deprivation and grievances (Cederman et al., 2013; Pearlman, 2013). While political science research has mainly focused on emotions that trigger political violence, criminologists often refer to emotions as source of restraint. Social concern, including care for others’ welfare, inhibits crime, as one of the creators of general strain theory argues (Agnew, 2014).

I use the typology of moral emotions introduced by Jonathan Haidt (2003). He distinguishes, among others, between emotions that express condemnation – contempt, anger, disgust (Rozin et al., 1999) – and emotions that express suffering for others, including compassion.^3^ Other-suffering emotions for potential targets of violence are an expression of disapprobation of violence against them. If moral salience activates the barriers to violence, it should thus be expressed in elevated levels of other-suffering emotions like compassion. The opposite should hold for other-condemning emotions for targets of violence, like anger or contempt, which indicate favorability to violence. The effects of moral salience on emotional favorability to violence are summarized in the next hypothesis:

Hypothesis 1b: Moral salience produces emotional disapprobation of violence.

### Group-Oriented Moral Beliefs and Collective Violence

The second logic of moral influence draws on moral pluralism, where right and wrong are not universal (Graham et al., 2011). In this understanding, moral beliefs are shaped by context, developed in response to idiosyncratic social norms, and constantly refined by the individuals who hold them. I do not examine this process, but the effect of holding certain moral beliefs on favorability to violence.

What kind of moral beliefs make individuals more inclined to favor collective violence? Given that humans have a general aversion to violence (Cushman et al., 2012), violence requires motivation. The key insight is that a morality of harm avoidance – the defining aspect of the moral domain according to the above moral salience logic – must be overturned. As exception, the infliction of harm on others is generally accepted in situations of self-defense. From a Western moral perspective, it is further permissible to use violence in war if harm is proportional and targets individuals who contribute to war (Dill, 2019; Dill & Schubiger, 2021).

More far-reaching views are though common across the globe. For example, for someone who regards their family as central good, violence may be a moral obligation in a situation of threat to their family, in extreme cases including blood revenge (Stein, 2019). Soldiers believe it is right to use violence in defense of their nation if their superiors demand them to do so, sometimes leading to “crimes of obedience” (Kelman & Hamilton, 1989). And certain religious doctrines may command people to use violence against those who violate the purity of religious rites (Moore, 2000).

These examples relate to a morality of loyalty (and group belonging), authority (and obedience to leaders), and purity (and the defense of sacred values). Beliefs based on these three moral foundations – Haidt and colleagues call them “binding”^4^ moral foundations (Graham et al., 2011; Haidt, 2013) – have the potential to overturn the “individualizing” harm avoidance and fairness^5^ concerns. Hence, distinct from the above proponents of moral restraint, the motivation for violence can come from within the moral domain.^6^ Beliefs based on binding moral foundations can make individuals feel morally obliged to act in favor of group values (Hoover et al., 2021; Janoff-Bulman & Carnes, 2013). Violence is then effectively seen as moral or legitimate (Fiske & Rai, 2014). Previous studies have focused on the relationship of specific moral foundations and support for violence, like authority-related values and torture (Benjamin, 2016), loyalty and inter-group violence (Cohen et al., 2006), as well as harm avoidance and military intervention (Kertzer et al., 2014; Kreps & Maxey, 2018).

I anticipate that the largest variation across individuals and communities concerns the relative importance assigned to binding as opposed to individualizing beliefs, which I call group-oriented moral beliefs. If binding beliefs are considered more important, inflicting harm may be seen as necessary.^7^ For example, an excluded group may use violence to achieve the greater good of political participation (as in rebellions), citizens may accept violence to establish order (as in counterterrorism policy), and religious groups may use violence to defend their practices (as in sectarian riots). In line with a prescriptive function of morality, which tells us what we should do (Janoff-Bulman & Carnes, 2013), I deduce the following hypothesis:

Hypothesis 2a: Group-oriented moral beliefs increase individual favorability to collective violence.

Similar to moral salience, group-oriented morals should be accompanied by relevant emotions, as moral judgment contains an emotional component (Prinz, 2009). Following the opposite logic as above, I expect individuals embracing group-oriented moral beliefs to display less other-suffering emotions for potential targets of violence who violated some rule, hence less compassion. Also, they should display more other-condemning emotions for the targets of violence, such as anger, exactly because they may see them as threat to their group values. The effects of group-oriented moral beliefs on emotional favorability to violence are summarized in the last hypothesis:

Hypothesis 2b: Group-oriented moral beliefs produce emotional approbation of collective violence.

## Research Strategy

### Lynching as Form of Collective Violence

For empirical analysis, I focus on lynching in Mexico City. By lynching, I mean *publicly displayed physical violence executed by a group of civilians against alleged wrongdoers*. While I do not intend to give a comprehensive explanation of this phenomenon, it can help us understand how moral beliefs influence favorability to collective violence for two reasons.

First, lynching provides a proximate outcome. Most forms of collective violence are rare, involve a small fraction of the population, are perpetrated by clandestine organizations, or determined by a small elite. These attributes make it hard to empirically identify the explanatory weight of moral beliefs. Lynching in Mexico City is so common that virtually everybody knows about it, many have observed it directly (roughly 23% according to my survey) and a considerable proportion of the population has participated in at least a “mild” form of lynching (10%). Studying a proximate form of collective violence allows us to examine participation, in addition to support. Previous studies have often assumed that support for violence is a gateway to actual violence. This is a strong and usually untested assumption. In this study, we can test it.

Second, lynching is susceptible to moral considerations. Lynching participants often claim to defend their community, provide rightful punishment, deter future criminals, and root out religiously impure practices (Gamallo, 2020; Godoy, 2006; Jung & Cohen, 2020; Kloppe-Santamaría, 2020; Nussio & Clayton, 2023). Such public good-oriented motivations suggest that morality is relevant for lynching. Selective incentives (Olson, 1965), organizational constraints (Tilly, 2003), and theories about ascribed identity (Sen, 2007) have little explanatory power for lynching in Mexico. Mob participants do not receive material benefits, are usually not part of an organization,^8^ and, in the context of Mexico City, belong to the same ethnic groups and social class as their targets (Zizumbo-Colunga, 2015, p. 5).

### Mexico City as Study Case

Studies on moral values and collective violence often compare across cultural contexts (e.g. Cousar et al., 2021; Inglehart et al., 2015; Karstedt, 2006). In such cross-cultural comparisons, the impact of moral beliefs may, however, be confounded with other conditions. For this reason, I focus on one context: Mexico City.

**Figure 1.**
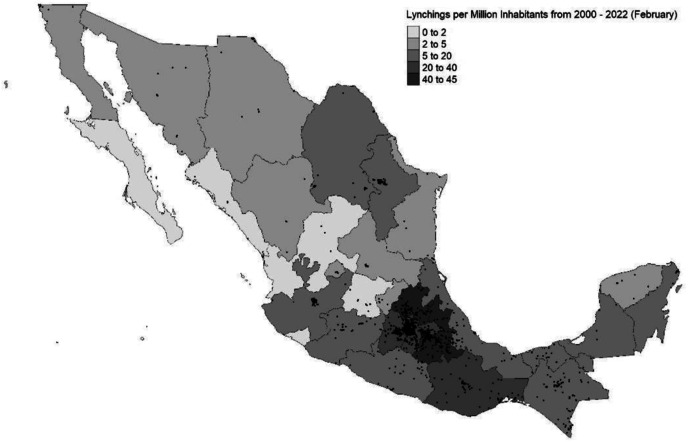
The distribution of lynching events in Mexico based on newspaper data.

Mexico City and its surrounding areas are a hot spot for lynching violence. This geographic concentration has been noted in historical studies on the first half of the 20^th^ century (Kloppe-Santamaría, 2020), and suggests that structural conditions related to community characteristics, a divide between state and society, and socioeconomic conditions account for it (Rodríguez Guillén & Veloz Ávila, 2019). Figure 1 shows the distribution of lynching events (black dots) reported in an original newspaper-based dataset (see A2) (Nussio & Clayton, 2022). As any newspaper-based violence data, this dataset underreports the actual number of lynchings, but it provides a sense of general tendencies. Official data on lynching in Mexico does not exist, as lynching is not defined as a crime in the penal code.

From 2000 to February 2022, newspapers reported 318 lynching events in Mexico City, with increasing frequency since 2018, despite reduced levels of homicidal violence in the same period. Lynchings respond to localized dynamics. They are most often triggered by thefts and amount to collective beatings. In Mexico City, they are particularly common in Southern peri-urban areas, and in marginalized areas of the city center and Iztapalapa. While the police often intervene to stop lynching, mob participants are rarely arrested, whereas the alleged wrongdoers who are targeted by the mob are often rescued and taken into custody.

The territorial division of Mexico City facilitates analysis sensitive to micro-level contexts. Mexico City is divided into roughly 1800 *colonias* (neighborhoods). Residents know in which colonia they live (it is part of their address) and it provides a relatable small-scale unit. I use colonias for sampling purposes and for analysis (see A3). By comparing individuals within colonias (using fixed effects), I keep contextual conditions constant across individuals. I further analyze whether the past occurrence of lynchings in a colonia makes any difference – it does not.

### Survey Design

In collaboration with a Mexican survey firm, I conducted a face-to-face survey representative of the adult population of Mexico City in February 2022, using a multi-stage sampling procedure (see A1) (Nussio & Parás, 2022). First, 340 colonias were selected using probability proportional to size sampling (Skinner, 2016), thus representing Mexico City. Second, enumerators selected a random initial location within the colonia and proceeded with a random walk, targeting 6 households. This resulted in a sample of 2183 participants (some colonias were oversampled).

After extensive piloting (see A1), the questionnaire covered demographics, social behavior, victimization, and questions about household surroundings (see A6). Questions about morality and lynching are discussed below.

A survey about violence requires sound ethical considerations (see A4). Particular attention was dedicated to legal implications, psychological distress, safety risks, and data protection. I further ensured full confidentiality and did not compensate participants financially. A Mexican legal counsellor was consulted and the university ethics board approved the procedures.

### Measurement of Lynching

The main dependent variables of hypotheses 1a and 2a concern favorability to lynching. In Mexico and elsewhere, the term lynching is often used as metaphor – Waldrep (2006, p. xvii) calls it a “rhetorical dagger” – for example to refer to harassment on social media, which would not fall under this article’s definition of lynching. I therefore abstain from using the term lynching in the questionnaire and ask a series of questions referring to a vignette describing a typical incident (adapted from CNDH, 2019, p. 78): *“A thief assaults a lady on the street. Using a knife, he takes her belongings and escapes. After the robbery, a passer-by manages to take away the thief’s knife and subdues the thief. In this moment, a large number of people gather, insult, and punish the thief.”*

The design of this vignette draws on the newspaper-based event dataset. In 60% of the cases recorded in Mexico, mobs target a single person; in 95% the main targeted person is male; 65% of newspaper recorded lynchings occur in response to alleged theft; and in 68% of the cases, the targeted person receives at least a collective beating (14% have a fatal outcome). The vignette presents the modal type of lynching in Mexico and is thus a valid representation.

After introducing the vignette, the questionnaire contains a series of support for lynching questions (measured on a four-point Likert scale), including: agreement with lynching; willingness to stand by and witness a lynching; willingness to personally participate in a lynching; neighbors’ encouragement of one’s participation; willingness to call the police to denounce the perpetrators (negatively coded), and agreement with sentencing the perpetrators (negatively coded). These items are analyzed individually and aggregated to an additive index (Cronbach alpha: .63). Due to a relatively low scale reliability of the easily interpretable additive index, all analyses including a support for lynching index are replicated with the first component derived from the same items using principal component analysis – results are consistent.

In addition, the questionnaire contains two behavioral indicators, which are analyzed separately: standing by and witnessing a lynching, and participation in lynching. Support and behavior items are correlated: willingness to participate in the future is correlated with agreement with lynching (Pearson’s correlation: .48) and with past participation in lynching (.25) (A5.5). While 45.2% state that they would participate in the future, 9.6% admit having participated in the past.

When asking about violence in surveys, people tend to give responses that are not truthful. The mild wording of the vignette should limit this possibility. The questionnaire does not describe an extreme form of violence, which reduces the participants’ willingness to report truthful answers (Westwood et al., 2022), and may have reduced affirmative answers to a negligibly small percentage, hampering statistical analysis. To make the vignette more vivid, I assigned a sex to the involved persons, including a male assailant of a female victim. This common combination of male and female protagonists plays into prevalent gender norms that make it easier for respondents to support the protection of a vulnerable victim (Kreft & Agerberg, 2023). It may thus raise the likelihood of affirmative answers.

The reliability of the question most sensitive to social desirability bias (participation in lynching) can be assessed with three previous surveys (A1). First, I conducted two anonymous online pilot surveys. Online surveys are less susceptible to social desirability bias (Kreuter et al., 2008). Using the Qualtrics platform^9^ and restricting the sample selection to Mexico City (*N* = 522 and *N* = 300), 7.3% and 8% respectively answered that they had participated in a lynching-style incident, thus slightly below the 9.6% in our face-to-face sample.^10^ Second, an identical vignette-based question was included in a survey representative of the whole of Mexico in November 2021 (*N* = 1019), conducted by the same survey firm. In this survey, 10.3% of participants stated that they had participated, close to our estimate for Mexico City. Furthermore, given that most respondents in the Mexico City survey agree with the perpetrators (71.3%), social desirability seems less problematic.

Is it ethical to ask participants about favorability to lynching? I followed guidelines about surveys on violence and victimization (UNODC, 2010). Also, I consulted a Mexican legal counsellor to make sure that the participants would not have to fear legal consequences, as Mexico mandates witnesses to report crimes to authorities. I thus worded the vignette in a way that would not describe a crime according to the Mexican penal code, using the non-legal term “punish” (*castigar*). Given the commonality of lynching in Mexico City, the implications of the term “punish” are clear for local respondents.

### Measurement of Moral Emotions

To capture moral emotions (see hypotheses 1b and 2b), I focus on other-suffering and other-condemning emotions (Haidt, 2003; see also Prinz, 2009). Moral emotions need to be elicited in response to some action. I therefore use the same vignette as for the lynching questions (about a thief being punished by a group of passers-by) and ask respondents about what emotions they principally felt when thinking about the thief – the target of violence.

Enumerators were instructed to note one principal emotion for each respondent. Answers were then hand-coded into dummy variables of *other-condemning emotions* (including mainly anger, but also indignation, disgust, contempt, and hate), and *other-suffering emotions* (including expressions of compassion such as empathy, pity, mercy, worry, and sadness). Amoral and unclear emotions (like fear, indifference, impotence, surprise, doubt, insecurity) were coded as zero. Roughly 43% expressed clearly identifiable condemning emotions for the thief. Approximately 17% expressed other-suffering emotions for the thief. Tellingly, some respondents used the opportunity of an open question to express their desire to kill the thief rather than to state an emotion.

### Moral Priming Experiment

To examine the logic of moral salience, I designed a priming experiment.^11^ Reminding people of their moral beliefs is an appropriate strategy to heighten their salience. The experimental treatment consists in asking participants about moral questions drawn mainly from the moral foundations questionnaire (MFQ) (Graham et al., 2011) – see below. For example, whether it is important to treat people equally, respect authority, show loyalty with their group; and whether they agree that it is never correct to kill somebody, that impure things are bad, and whether certain forms of violence are agreeable (such as torturing suspected terrorists). In total, they are confronted with 38 such questions (full questionnaire: A6). Participants thus think about their own moral beliefs for about five to 10 minutes. At no point do the enumerators suggest what they consider right and wrong.

**Figure 2.**
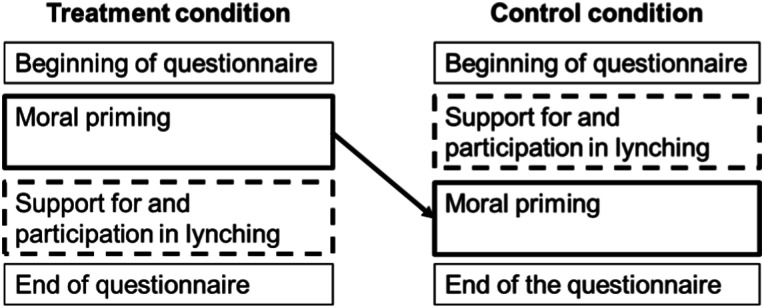
Moral priming experiment.

This block of questions appears randomly before (treatment) or after (control) introducing the lynching questions (Figure 2).^12^ Hence, half of the survey participants were primed to think about moral beliefs before they answered questions about lynching and the other half not (A5.3 shows covariate balance).^13^ It is not possible to evaluate the separate effects of priming one or the other moral belief, an approach that should be used in future studies.

To interpret this experiment correctly, the following assumptions must be met. First, causality needs to run into only one direction (from moral beliefs to lynching, and not from lynching to moral beliefs). Given that I measure both lynching support and moral beliefs, I can identify where changes come from. There are no changes in the answers to morality questions, but only in the lynching questions (A5.4 and A5.11). It is thus unlikely that the lynching questions affected answers to morality questions and that causality runs into the opposite direction.

Second, the moral priming does not influence answers only by inducing social desirability. If respondents show less support for lynching because they think that this is what the enumerators expect from them, lowered support amounts to cheap talk. To check for social desirability, I compare the answers for lynching support to answers for participation in lynching. If the moral priming influenced lynching support through social desirability, it should also influence responses to lynching participation. This is not the case (A5.10). It is thus unlikely that the results are driven solely by social desirability.

### Measurement of Group-Oriented Moral Beliefs

To measure group-oriented morals, the moral foundations questionnaire (MFQ) provides a useful tool (Graham et al., 2011). The MFQ includes six questions for each moral foundation (harm avoidance, fairness, loyalty, authority, and purity; see A6) using a 0 to 5 Likert scale. Harm avoidance and fairness are grouped under the label of individualizing foundations, whereas loyalty, authority and purity are grouped as binding foundations. I use the difference between binding and individualizing morality items as main indicator for group-oriented moral beliefs. In addition to capturing the key conceptual insight of privileging group values over individual values, using the difference also accounts for an important source of systematic response bias. While responses to single items about moral beliefs are generally skewed towards high scores, the difference reproduces relevant variation between binding and individualizing items and is normally distributed.

The difference variable ranges from −2.9 to +1.9 with a mean of −.4. Individualizing values thus find on average more support than binding values. For comparison, with a mean of −.4 the sample of Mexico City residents is right in between what Graham et al. (2011; own calculations) call conservatives (+.1) and moderates (−.9) in their US sample (standard deviations are similar to the Mexico City sample). In addition to this combined measure, I also use separate indices of binding and individualizing values and find similar results (A5.17 and A5.18).^14^

I deliberately abstain from using an experimental approach to manipulate group-oriented moral beliefs. While it is possible to prime individuals for aspects of group-oriented morals (Hoover et al., 2021; e.g. Mooijman et al., 2018b), I see the content of moral beliefs as relatively stable.^15^ Furthermore, the chosen observational approach allows for the study of participation in addition to support.

### Analysis Procedures

Table 1 provides an overview of the analysis. To avoid cherry-picking “significant” results, I conduct all analyses using linear regression with two extreme modeling specifications using individuals as unit of analysis. The “simple” model includes a limited number of demographic control variables: sex (51% female), age (mean: 41), level of education, and wealth. The “demanding” model includes additional control variables, colonia level fixed effects, and robust standard errors clustered at the colonia level. Also, rather than singling out specific results, I focus on consistency across tests, which raises the bar for falsely rejecting null results.

**Table 1.** Overview of Analysis Strategy.

Theory	Hypothesis	Independent variable	Dependent variable	Analysis type
Moral salience	1a	Moral priming	Support for lynching	Experimental
	1b	Moral priming	Moral emotions	Experimental
Group-oriented moral beliefs	2a	Binding minus individualizing beliefs	Support for and participation in lynching	Observational
	2b	Binding minus individualizing beliefs	Moral emotions	Observational

Observational analysis does not allow for straightforward causal inference. However, the specification of the “demanding” model contains the risk of confounding by using colonia-level fixed effects which adjust for everything that is the same for individuals from the same neighborhood, including the location within Mexico City, general crime exposure, and lynching occurrences. Furthermore, I include additional individual level covariates (see A5): religious affiliation to account for traditional beliefs that may be confounded with moral beliefs (Bailey & Snedker, 2011; Cousar et al., 2021) – 63% indicate being catholic and 21% non-religious; employment status to account for time spent in the neighborhood – 61% indicate that they were working and 6% unemployed; participation in a fight to account for individual propensity to violence – 26% had participated in a fight; trust in government to account for one’s view of state authority (Levi, 1997; Nivette, 2016) – average of 3.9 on 1–7 scale; a variable to correct for systematic bias in responses to the questionnaire; and commercial use and public space cleanliness of the street block to account for within-colonia differences (Vilalta et al., 2020) (the last two coded by enumerators in situ).

## Findings

### Moral Salience

**Figure 3.**
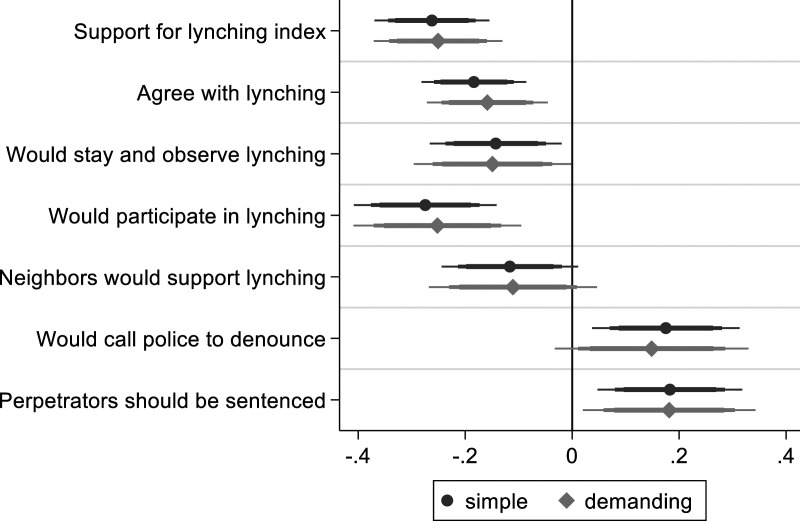
The effect of moral priming on lynching support. *Note*: OLS regression coefficients with 99, 95 and 90% confidence intervals. Simple model includes limited number of control variables. Demanding model with additional control variables, clustered standard errors and colonia fixed effects.

First, I examine whether the experimental moral priming reduces favorability to lynching, as argued in ***hypothesis 1a***. Figure 3 reports the main results (full regression table: A5.6 and A5.7). The dark gray coefficients represent a simple model including few demographic control variables. The more precisely estimated light gray coefficients represent a demanding model including additional control variables, clustered standard errors, and colonia-level fixed effects. The width of horizontal lines represents confidence intervals of 99, 95 and 90% respectively. If the intervals do not overlap with the vertical line drawn at 0, the coefficient, represented by circles and diamonds, is statistically distinguishable from 0 at the respective level of confidence.

I find reduced levels of support for lynching for the additive index and for all individual items measuring support. I also see increased agreement with the last two items capturing rejection of lynching: calling the police to denounce the perpetrators and sentencing the perpetrators. Most coefficients for support are between −.15 and −.25 on a four-point Likert scale. The substantively most important variable (willingness to participate) has the largest coefficient of −.25. Without accounting for co-variates, the average score of this variable decreases from 2.42 to 2.15.

In A5.10, I show that moral priming does not affect reporting of participation in lynching. This confirms that the results are not driven by social desirability bias. Furthermore, results for moral priming hold among both individuals with strong and weak group-oriented moral beliefs. This speaks to the universality of harm avoidance as restraining force. Individuals are susceptible to moral salience independent of the content of their moral beliefs. In sum, the presented evidence supports the main moral salience hypothesis.

**Figure 4.**
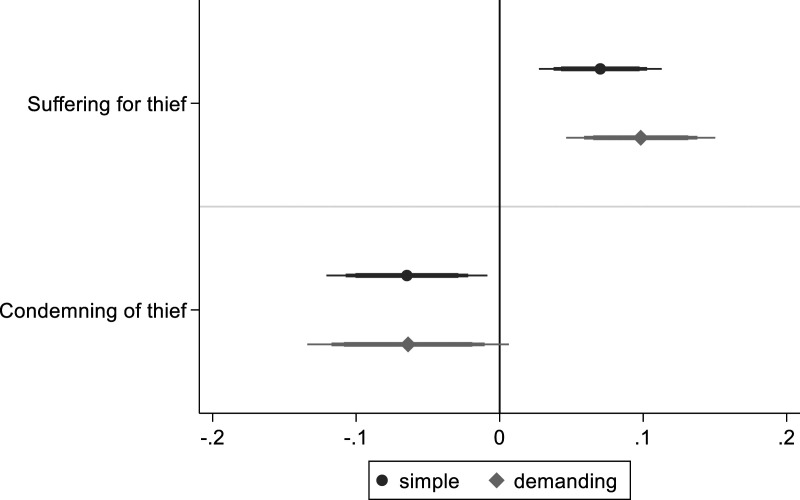
The effect of moral priming on moral emotions. *Note*: OLS regression coefficients with 99, 95 and 90% confidence intervals. Simple model includes limited number of control variables. Demanding model with additional control variables, clustered standard errors and colonia fixed effects.

According to ***hypothesis 1b***, moral salience should produce emotional disapprobation of violence. Figure 4 shows that respondents primed for morality have more other-suffering emotions, i.e. compassion, for the target of violence (full regression tables: A5.8 and A5.9). Without accounting for covariates, such emotions increase from 13.7% to 20.6%. In addition, they are less condemning of the thief. As expected, moral salience is accompanied by less violence-approving moral emotions, consistent with an understanding of moral judgment as containing an emotional dimension.

In additional correlational analysis (A5.12), moral emotions are strongly related to support for lynching. Other-suffering emotions for the target are associated with less support for lynching, whereas other-condemning emotions are associated with increased support. These results underline that moral emotions are intricately related with support for violence and accompany moral judgement. Favorability to violence can be expressed as either attitudinal support or emotional approbation.

### Group-Oriented Moral Beliefs

**Figure 5.**
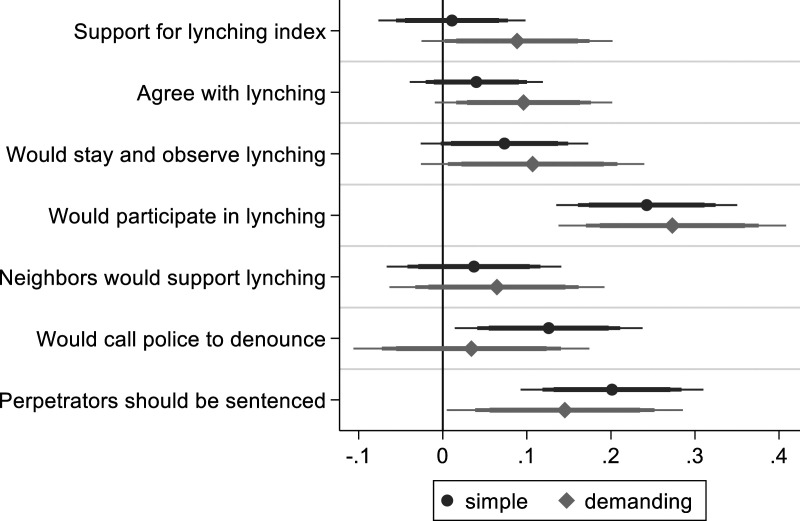
Group-oriented moral beliefs and lynching support. *Note*: OLS regression coefficients with 99, 95 and 90% confidence intervals. Simple model includes limited number of control variables. Demanding model with additional control variables, clustered standard errors and colonia fixed effects.

Next, I examine whether a variable that indicates privileging of binding over individualizing morals increases favorability to collective violence, as argued in ***hypothesis 2a***. Figure 5 shows a positive association of group-oriented morals with all items indicating support for lynching, although some coefficients are indistinguishable from 0, especially in the “simple” specification (which does not account for most sources of confounding) (full regression tables: A5.13 and A5.14). The substantively most important item (willingness to participate) shows the largest coefficient. For each 1-point increase in group-oriented moral beliefs (ranging from −2.9 to +1.9), there is a .27 increase in willingness of future participation (A5.14). Contrary to expectations, group-oriented beliefs are positively associated with items capturing rejection of lynching, including calling the police and sentencing the perpetrators. The moral foundations questionnaire includes moral beliefs related to respect for authority and patriotic loyalty as part of its binding foundations. This may explain why group orientation is associated with increased collaboration with national authorities, especially in the “simple” model before accounting for trust in government.

**Figure 6.**
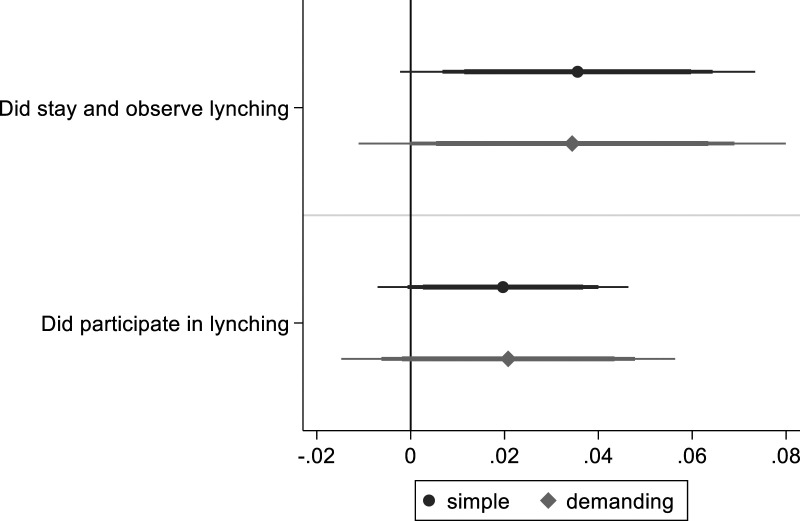
Group-oriented moral beliefs and lynching behavior. *Note*: OLS regression coefficients with 99, 95 and 90% confidence intervals. Simple model includes limited number of control variables. Demanding model with additional control variables, clustered standard errors and colonia fixed effects.

One advantage of observational analysis is that I can analyze lynching participation in addition to support. For this analysis, I assume that the content of moral beliefs is relatively stable, given that the participation variable refers to the past. To the extent that readers share this plausible assumption (Haidt, 2013), the following analysis provides useful information. Figure 6 shows that group orientation is positively associated with staying and observing a lynching incident, and with participation (full regression tables: A5.15 and A5.16). The estimate for participation in the demanding model is though not statistically distinguishable from 0 with a high level of confidence.

Given the limitations of observational analysis, I provide additional analysis to assess the robustness of the relationship between group-oriented moral beliefs and lynching favorability. First, one could argue that individualizing beliefs, especially harm avoidance, motivate individuals not to engage in violence, much like for moral salience. Hence, the effect of group-oriented morals would mainly be driven by individualizing beliefs. I show results for binding morality and individualizing morality separately (A5.17 and A5.18). Results indicate that binding morality is positively related with support and participation, and individualizing morality negatively related to both. This suggests that the positive relationship of group-oriented beliefs comes from both and that the difference between binding and individualizing values is most decisive.

Second, I show the relationship of group-oriented morals with non-equivalent outcomes, including non-violent collective action like participation in community activities, protest, and religious activities. Except for religious participation, group-oriented beliefs are not related to social participation, suggesting that the link between group orientation and lynching favorability is not just an artifact of generally increased participation (A5.19).

Third, I replicate the analysis using colonias instead of individuals as units of analysis (*N* = 340) and drawing on an original dataset with additional colonia-level characteristics (A3).^16^ In theory, a colonia with – on average – higher levels of group-oriented morals should also see more lynching events, given that such beliefs may facilitate cooperation for the enactment of actual lynchings. While this analysis set-up is less robust than individual level analysis, I find suggestive evidence for a positive relationship between group-oriented moral beliefs aggregated to the colonia level and independently measured survey-based and newspaper-based indicators of lynching events (A5.20).

Fourth, I assess the sensitivity of key findings using Oster’s approach (Oster, 2019). This approach relies on the insight that the stability of coefficients across models with additional control variables and simultaneous changes in model fit (*R*^2^) indicate limited omitted variable bias. Following recommended procedures, I find that the most important results reported in observational analysis (referring to whether an individual would participate in lynching or has participated in lynching) are not sensitive to omitted variable bias (A5.24).

Fifth, I assess heterogeneous effects. In descriptive terms, females are generally less and crime victims more favorable to lynching, while respondents from colonias with past lynching are similarly favorable to lynching than respondents from colonias without. However, I do not find relevant differences in associations to group-oriented moral beliefs (or moral salience) when comparing these groups. The results are thus consistent across subgroups.

**Figure 7.**
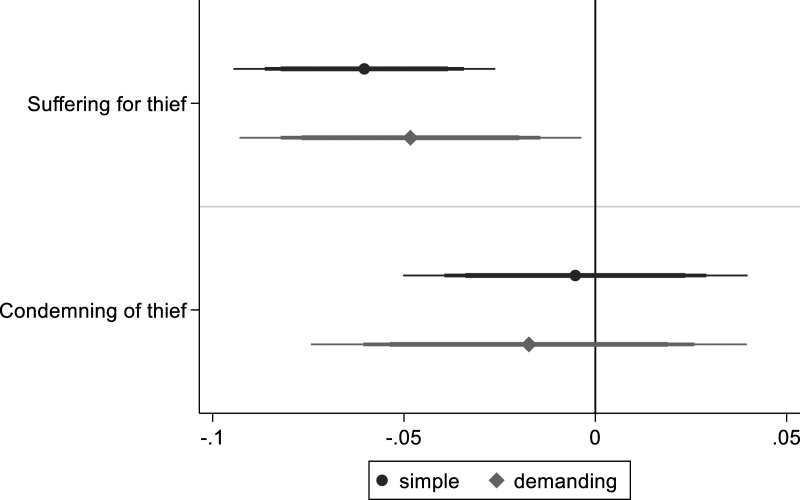
Group-oriented moral beliefs and moral emotions. *Note*: OLS regression coefficients with 99, 95 and 90% confidence intervals. Simple model includes limited number of control variables. Demanding model with additional control variables, clustered standard errors and colonia fixed effects.

According to ***hypothesis 2b***, group-oriented moral beliefs should produce emotional approbation of collective violence. Figure 7 shows that compassion for the target of violence is reduced for individuals who embrace binding values more than individualizing values (full regression tables: A5.21 and A5.22).^17^ Hence, group-oriented morals are accompanied by less other-suffering emotions, emphasizing the emotional dimension of moral judgment. Contrary to expectations, group-oriented moral beliefs are not associated with condemnation of the thief.

As described above, other-suffering emotions are negatively related to support for lynching. In additional analysis, a similar relationship holds for lynching behavior. Suffering emotions are related to less participation and condemning emotions to more participation in lynching (A5.23), again underlining the intricate relationship between moral judgment and moral emotions, and their behavioral implications. Overall, I find substantial observational evidence in line with group-oriented moral beliefs favoring collective violence.

## Conclusions

In this article, I argue that moral beliefs influence favorability to collective violence depending on moral salience – whether moral beliefs are a relevant part of decision-making or not – and group-oriented moral beliefs – motivating individuals to overturn the harm avoidance principle. Survey evidence from Mexico City on support for and participation in lynching is largely in line with my hypotheses. I find that randomly assigned moral priming reduces favorability to lynching and stimulates the violence-averse emotion of compassion for the target of violence. Group-oriented moral beliefs are associated with more favorability to lynching and decreased levels of compassion. Group-oriented beliefs are further related to participation in lynching. Despite their origin in opposing views on morality (a universalist morality based on harm avoidance vs. moral pluralism), both logics of moral influence receive support.

These findings have implications for theory. They substantiate a micro-link between morality and collective violence, assumed in several macro-level theories but rarely tested (see also Kertzer, 2017). Moral beliefs do not just represent post-hoc justification but can influence individuals’ favorability to collective violence. Thinking about broader social implications, moral salience and group-oriented morals may in part explain the long-term decline in violence encountered in previous research, through a generalized tendency towards individualizing values in Western society (Eisner, 2014; Inglehart et al., 2015). Furthermore, I show how moral judgement has an emotional dimension, establishing a link to recent literature on emotions and conflict (Pearlman, 2013; Penic et al., 2018; Stekelenburg, 2017).

Readers may claim that, in contradiction to my theorizing, moral beliefs focusing on individualizing goods rather than groups can also incite violence. Examples include activism for social justice turned violent (Zaal et al., 2011) or liberal governments using violence to live up to their responsibility to protect, in favor of individualizing values. This is certainly true. Supporters of any morality, including those fighting for justice, may conclude that they can only defend their values with violence. The philosophical debate about just war is a good example (Walzer, 2015). However, until further research can demonstrate otherwise, I believe that individualizing values of harm avoidance but also fairness are more related to non-violent strategies (Dahlum et al., 2022). In the case of lynching, mob participants often claim to act in the name of justice (Goldstein, 2003), which is part of the moral foundation of fairness. While this motivation is certainly believable, their concern for justice is though not what distinguishes them from non-participants, perhaps because justice concerns are largely universal (Kohlberg, 1981).

Readers may also claim that different kinds of moralities have different social benefits. For example, communities with a group-oriented morality may prevent certain forms of violence, like petty crime and suicide, as their members are concerned about losing group approval (Rottman et al., 2014; Silver & Silver, 2020). In addition, individuals subscribing to group-oriented moral beliefs may be more compassionate and altruistic in local everyday interaction. However, their compassion may be limited by group considerations, as in communities characterized by parochial altruism. Group-oriented moral beliefs are then a resource for group identification which facilitates collective action, including violent collective action (Littman & Paluck, 2015).

In this study, I focus on lynching in Mexico City, which opened a window into how morality influences favorability to collective violence. First, studying individuals situated in their respective neighborhoods made it possible to address key sources of confounding, a limitation of cross-cultural research on the topic (Karstedt, 2001). Second, centering the study on lynching made it possible to examine a form of collective violence that participants know from first-hand experience. However, readers may be concerned about the generalizability of the presented findings.

What can lynching tell us about other forms of collective violence? One may argue that morality is particularly important for spontaneous mob violence, as it compensates for the lack of organization and thus facilitates collective action. However, should violent organizations not also try to tap into the power of morality to support cooperation? This rhetorical question suggests that morality plays a role beyond mob violence but studying morality in the context of organizations is hard. Moral beliefs are built into organizational culture, ideology, and identity. This is exactly the reason why lynching is an appropriate study object. The unorganized nature of lynching, though, limits inferences to more organized forms of collective violence.

Others may think that moral beliefs are most relevant in the context of weak or illegitimate states, as they compensate for deficiencies in the rule of law. Previous studies find that related forms of informal justice, sometimes involving violence, may substitute formal institutions in weak states (R. Blair et al., 2018). While I agree with this view, few polities are able to convince all their citizens of their legitimacy (Levi, 1997), and moral beliefs are thus a powerful guide for action – including collective violence – even in developed democracies. The storming of the United States Capitol on 6 January 2021 is a clear example.

When thinking about generalizability, interpreting lynching as punishment violence is illuminating (Garland, 1990). Previous research shows that the desire to punish explains support for war and torture in the US (Liberman, 2014; Liberman & Skitka, 2017; Stein, 2019). From the Israel-Palestine conflict, over the Northern Irish post-conflict period to the Mexican war on drugs, punishment is a key function of violence and is accompanied by the dominant moral emotion of anger (Asif, 2022; García-Ponce et al., 2023; Rickard & Bakke, 2021; Zeitzoff, 2014). To the extent that collective violence is used for the purpose of punishment (Fiske & Rai, 2014), we can draw inferences from lynching to other forms of collective violence. Moral salience may, for instance, increase levels of compassion for potential targets of violence and reduce a desire for punishment.

Lynching is more common than Western audiences assume. It is a prevalent form of violence across Latin America and in some of the most populated countries around the world, including India, Indonesia, South Africa and Nigeria (Jung & Cohen, 2020). It is a symptom of a broken relationship between the state and society, and thus deserves more attention from political science. Future studies should also expand into other forms of collective violence and geographic regions to examine the scope of moral salience and group-oriented moral beliefs. However, without taking context seriously, they will make little headway towards improved understanding. Collective violence is always contextually situated and corresponds to repertoires that are not universal (Gutiérrez-Sanín & Wood, 2017). Also, looking into the moral beliefs surrounding violent organizations, such as rebels, gangs and hooligans, is a challenging but fruitful avenue, and may complement research on ideology (Maynard, 2019) and identity (Littman & Paluck, 2015).

The present study further provides lessons for practitioners of violence prevention. For example, discussing morality is already part of cognitive behavioral therapy for at-risk youth (Heller et al., 2015). Such interventions though only work if executed by trusted authorities (G. Blair et al., 2021). The example of historical lynching in the US South shows that moral values can influence the use of violence, as increased publicity of racial killings led to counternarratives, ultimately contributing to the demise of lynching (Weaver, 2019). While training the “moral muscle” may reap some benefits, this study’s findings do, however, not support the naïve conclusion that morality is the magic bullet against violence.

## Supplemental Material

Supplemental Material - How Moral Beliefs Influence Collective Violence. Evidence From Lynching in Mexico Supplemental Material for How Moral Beliefs Influence Collective Violence. Evidence From Lynching in Mexico by Enzo Nussio in Comparative Political Studies.

## Data Availability

Replication materials can be found at (Nussio, 2023).

## Notes

1. Previous studies have manipulated aspects of group-oriented morality (Hoover et al., 2021; Mooijman et al., 2018b).

2. Replication materials and code can be found at (Nussio, 2023).

3. Bloom (2016) distinguishes between emotional empathy (coming to experience the world as you think someone else does) and cognitive empathy (understanding the pain of someone else, without feeling it oneself). I view compassion as including both.

4. Binding and individualizing moral foundations are similar to collectivist and individualist values (Oyserman et al., 2002).

5. Fairness is usually classified as individualizing foundation. Connected to identity, however, strong fairness susceptibility may fuel grievances and promote certain types of collective violence (Cederman et al., 2013).

6. Interestingly, moral disengagement theory includes similar resources to overcome barriers to harm, including loyalty and authority, but locates them outside the moral domain (Bandura, 2016, p. 30).

7. This has an implication for the moral salience argument, as raising the salience of group-oriented morals could increase favorability to collective violence. I examine this implication by comparing the effects of raising moral salience in subgroups of individuals with high and low levels of group orientation. I do not find any differences.

8. This distinguishes lynching in Mexico City from more organized forms of vigilantism (Bateson, 2021; Moncada, 2022).

9. Online panels differ from the general population (Huff & Tingley, 2015). Hence, these samples have exploratory character and are not used for analysis.

10. I also tested a list experiment in the pilot (García-Sánchez & Queirolo, 2021). It did not reveal a different proportion of affirmative answers and was thus not used in the face-to-face survey.

11. This experiment was not pre-registered, and results have to be seen as exploratory.

12. Due to a synchronyzing error of the survey software, it was impossible to identify the moral priming condition for 117 individuals. These individuals are not statistically different from the remaining sample.

13. This ordering design was inspired by Oyserman et al. (1998).

14. I also use an indicator for the difference between binding values and harm avoidance (without including fairness). Results remain consistent.

15. Stability does not imply that moral beliefs are heritable, unchangeable traits, as Smith et al. (2017) point out.

16. Lynching event data can be accessed through this interactive app: https://css-ethz.github.io/lyla/.

17. Results remain substantively identical using logistic regression modeling.
