# Supplementary material for: How Moral Beliefs Influence Collective Violence. Evidence From Lynching in Mexico
Source: Comp Polit Stud. 2023 Dec 27;58(1):43–77. doi: 10.1177/00104140231223747 (PMC11573652; doi:10.1177/00104140231223747)
Supplement: Supplemental Material - How Moral Beliefs Influence Collective Violence. Evidence From Lynching in Mexico [file sj-pdf-1-cps-10.1177_00104140231223747.pdf]

Appendix to "How Moral Beliefs Influence Collective Violence. Evidence from Lynching in Mexico"

Enzo Nussio, Center for Security Studies, ETH Zurich

Replication materials and code can be found at:
Nussio, Enzo. 2023. "Replication Data for: How Moral Beliefs Influence Collective Violence. Evidence from Lynching in Mexico." Harvard Dataverse. https://doi.org/10.7910/DVN/X6E6XC.

Contents

A1. Survey procedures
A2. Newspaper-based lynching event dataset: short description
A3. Colonia-level dataset: short description
A4. Ethical considerations
A5. Additional statistical analyses
A6. Links to full survey questionnaire and consent sheet
A7. References

A1. Survey procedures

Additional description of the questionnaire

The survey questionnaire was prepared over the course of one and a half years mainly in collaboration with the Mexican survey firm Data OPM and research assistants. The questionnaire drew in part on previously tested questions used in the Latin American Public Opinion Project surveys in Mexico, the largest survey program in Latin America.[1] Additional questions were developed independently in collaboration with the survey firm. For full questionnaire, see A6.

The vignette describing a typical lynching incident was inspired by a similar wording used in the survey of the Comisión Nacional de Derechos Humanos on lynching in Mexico (CNDH 2019). It is important to mention that the item about participation in lynching comes after the item about witnessing and standing by. Hence, it is clear from the question ordering that participation goes beyond mere bystanding.

Questions on morality were adapted from the Spanish version of the Moral Foundations Questionnaire.[2] Some of the language was adapted to the Mexican context.

The items on agreement with other forms of violence were inspired by a series of related studies (Smith et al. 2014; Cohen, Montoya, and Insko 2006; Slovic et al. 2020; Herrero, Rodríguez, and Torres 2017; Sundberg 2014; Hornor et al. 2015). The item about removing a corrupt mayor was inspired by a historical novel (Benítez 1992).

To avoid ordering effects within blocks of questions, we fully randomized the ordering of questions that belonged to the same battery of questions (including questions about morality and different forms of violence).

The questionnaire also includes an additional experiment (related to victimization), that will be reported in a future publication.

Piloting and questionnaire development

The long period of survey preparation was in part due to piloting. A different version of the survey questionnaire was piloted twice on the Qualtrics Platform restricting the sample to residents of Mexico City. Once from June 25 to July 5, 2021 (N=300). We do not refer to this pilot in the main text

[1] See https://www.vanderbilt.edu/lapop/
[2] See https://moralfoundations.org/questionnaires/

as the questionnaire changed drastically as a result of it. The second pilot was fielded from November 8 to 25, 2021 (N=522). This pilot was also used to examine a list experiment for the participation in lynching question (García-Sánchez and Queirolo 2020).[3] Proportions from the list experiment and the direct question did not differ significantly, which is why we abstained from including the list experiment in the final questionnaire.

In the second pilot, we also tested an experimental treatment for group-oriented morals. We used a picture and description of the 2017 earthquake in Mexico to prime loyalty values. However, while the prime generated solidarity, it also strengthened a morality of harm avoidance. We therefore discarded the use of an experimental treatment for binding morality, and opted for observational analysis. Other options adapted from previous studies (Mooijman et al. 2018) focused too specifically on one of the binding foundations and were discarded due to conceptual concerns.

As a result of the second pilot, we also changed the questions regarding moral emotions. We previously used a set of fixed answer options. The face-to-face survey allowed us to use an open answer question more effectively, given that the enumerators had clear instructions about how to record moral emotions.

In addition, the vignette including three key questions of the questionnaire (agreement with lynching, participation in lynching and knowing about lynching in community) was fielded in November 2021 in a face-to-face Omnibus survey representative of the Mexican adult population (N=1019).

Sampling

The survey was designed to allow for both individual and aggregate-level analysis (aggregate-level analysis will be the focus of future publications). For that reason, we targeted an unusually high number of primary sampling units (colonias). The targeted number of 340 units was a compromise between sufficient dispersion across the city and sufficient numbers of interviewees within each unit (at least 6). The 6 individuals in each unit were block-randomized to the three victimization treatment conditions (the second survey experiment that will be reported in an additional paper), and randomly assigned to the moral priming experiment.

The 340 colonias were selected by the principal investigators using probability proportional to size (PPS) sampling (Skinner 2016) without replacement, implemented in Stata 16. There is no readily available information on colonia-level population. We therefore created a colonia-level indicator for population size based on census information for the street block, or "manzana", level (see Colonia-level dataset below) (Vilalta, Muggah, and Fondevila 2020). We also selected a secondary set of 70 replacement colonias using the same procedure, to allow for quick replacement in case the enumerators faced security problems or could not access buildings. In 19 colonias, interviews had to be complemented in directly adjacent areas or colonias had to be replaced completely (using colonias from the same borough). The main reason was restricted access to gated communities in wealthy neighborhoods. One colonia had to be replaced due to security concerns for the enumerators, a much lower number than initially expected.

The selection of households within colonias was identical to the procedures used by the widely tested Latin American Public Opinion Project. First, enumerators identified the Northeastern corner of a randomly chosen starting street block (*manzana*) within the selected colonia. From this point, the enumerators followed a random walk procedure, contacting every fifth household around the street block. Then, they selected a further street block using the same procedure until they reached the targeted 6 interviews reflecting an equal amount of men and women, and an age distribution in line with census information. Given that a team of enumerators was working within the same colonia at the same time, certain colonias have slightly more than 6 interviews, but all have at least 6 interviews.

[3] The question of the list experiment was as follows. While a randomly selected half of the respondents received only 3 response options, the other half also received option 4: «Now, I am going to mention a series of [3/4] activities and I would like to know *how many* of them you have done in the past, not *which* ones. Simply add up the number of activities you have done, incidating a total number between 0 and [3/4]. At some point, have you (1) cleaned up a park in the neighborhood, (2) crossed the street when the traffic lights were red, (3) fixed the public lighting in your neighborhood [, (4) participated, with your neighbors, in the punishment of a thief.] How many of these activities have you ever done in the past? 0, 1, 2, 3 [, 4])

Survey fielding

The survey was implemented as a face-to-face survey due to the increased difficulty of reaching individuals by phone during the pandemic[4] and the limited possibilities in terms of geographically focused sampling in online surveys. Overall, the quality of a face-to-face survey was considered superior for our purposes.

A total of 61 previously trained enumerators conducted the survey between Febuary 9 and March 1, 2022. The non-response rate was 44%. After introducing themselves, the enumerators began with a short description of the survey and handed out a consent sheet, including contact information to the study organizers and psychological counselling (see Ethical Considerations below). The average duration of the interview was 19 minutes. The last block of questions was directed to the enumerators, who filled them out without the presence of the respondents.

In the field report of the survey firm, the enumerators noted the following difficulties: encountering male participants as they are often out of the household, and access to gated communities. Also, some questions had a difficult wording for participants with low levels of education. Advantages included the fact that the enumerators could present themselves as working for a Swiss university and the topic of the survey (insecurity), which is of general interest to residents of Mexico City.

Survey monitoring

The survey implementation was monitored by 12 supervisors, 3 auditors and 2 team coordinators, including in-person supervision of 15% of the sample and remote supervision of another 12%. The survey platform Survey To Go automatically recorded audio files of two short segments of the interview, without knowledge of the enumerators, to guarantee the effective application of the survey. Also, enumerators had to provide geographic and photographic information (not of the study participants) to show that they effectively conducted the interview where they were supposed to conduct the interview and at the time they were supposed to conduct the interview. Interviews with evidence that enumerators did not conduct the survey appropriately were removed. Also, unrealistically short interviews and interviews with overly fast progress from one question to the next were removed. Identical responses to a series of questions were taken as a potential warning sign.

[4] According to Mexican survey firms, non-response rates of phone surveys have increased considerably during the pandemic. In previous years, our survey partner needed roughly 25 calls for each completed survey; during the pandemic, they needed 130 calls for each completed survey.

A2. Newspaper-based lynching event dataset: short description

The newspaper-based lynching event dataset includes evidence of 2818 lynching incidents in Latin America between 2010 and 2019.[5] It is based on the manual coding of newspaper reports about lynching events contained in the Factiva global news monitoring and search engine. It is the first Latin America-wide lynching dataset spanning multiple years of observation, containing a wide set of variables (including a geo-coded location), and using a unified coding scheme and data source.[6]

We focus exclusively on newspapers for several reasons. First, for ethical reasons. Coding of written reports is less harmful for coders than being exposed to explicit and visual content of social media entries. Second, we believe that despite the limitations and biases of newspaper reporting, this source has clear benefits compared to other potential sources. Unlike social media, newspapers provide archives reaching back in time. Unlike survey-based measures, they provide broad geographic coverage and high information granularity. For these reasons, we chose to study lynchings across Latin America through newspaper reports. Official data on lynching are not available in any of the countries, as lynching is not codified as a crime in penal law.

While concerns about underreporting need to be taken into account, the data used for the present article gives broad orientation with regard to the geographic distribution of lynching in Mexico and across Mexico City.

[5] For Mexico, we extended the observation period to include the years 2000 to February 2022 (1859 lynching events).

[6] Detailed materials, including the codebook and the dataset itself, will soon be publicly available.

A3. Colonia-level dataset: short description

For analysis at the colonia level, we created our own dataset. It is mainly based on point data about crime (homicide and other types of crime), census data on population characteristics, and administrative data on geographic characteristics. These data were aggregated to the colonia level (N=1820). A large share of these data sources are freely available on the web.[7]

The population data was particularly important for the sampling of colonias. It is based on Census information about population numbers for each street block (manzana) in Mexico City. Given that some manzanas are split into different colonias, we assigned a population number proportional to the size of the manzana in each of the respective colonias. Due to this procedure, the population numbers at the colonia level must be seen as an approximation. However, they are precise enough to allow for sound population proportional to size sampling.

The same colonia dataset is also used for aggregate level analysis. However, this is only auxiliary analysis for the present study.

[7] See https://datos.cdmx.gob.mx

A4. Ethical considerations

In addition to the short references in the main paper, here I report in more detail on how I dealt with ethical challenges, focusing mainly on risk mitigation strategies. The Ethics Board of ETH Zurich approved the proposed procedures.

This study does not create health risks for any of the participants as we only collect information about their attitudes, beliefs, and narratives. Also, we do not foresee any societal-level or political risks stemming from this study. Similar research endeavors have been carried out in the past – for example a survey on lynching in Mexico (CNDH 2019). Thus, the wider public is aware of the topic and direct political consequences are unlikely.

Our project is about collective violence. In order to better understand why collective violence occurs, we ask participants about their experience with and participation in collective violence. This is an important undertaking as it can potentially help us identify violence prevention tools, but it also has implications for ethically sound research. We are highly aware of potential risks and abstain from exposing participants to any excessive risks. We specifically focus here on psychological distress, legal implications, security risks for researchers, risks from the Corona pandemic, and data protection, and discuss our strategies to mitigate these potential risks.

Psychological distress

After extended conversations with our local partners (Data OPM, legal counsellor and academic partners), we assess the risk of re-traumatization during face-to-face interviews to be minimal. While the term lynching evokes grisly associations among a Western audience who might think about brutal lynchings in the US, targeting African Americans in the 19th and early 20th century, Mexicans are constantly exposed to acts of self-justice, which usually amount to beatings of thieves, either in the news or through their own experience. This is also how we represent these events in our vignettes – we abstain from using the term lynching or “linchamiento”. For Mexicans, these events are normalized as well as surveys and studies about security-relevant questions. Hence, the risk of re-traumatization is minimal.

However, there may be rare cases in which study participants suffer emotional distress as a result of our surveys and interview questions. As a general stance, we avoid using value-laden language in our surveys and interviews. Also, we avoid talking about specific forms of violence that occurred during a lynching as this is not the main focus of our inquiry. This should generally help to limit the risk of psychological distress.

We provide phone numbers of official agencies and contacts of free psychological counselling to give study participants the possibility to choose options that are independent from us. Local partners have indicated that this is preferable to connecting them with a specific service, which may be seen suspiciously.

More specifically, in preparation of the surveys we further made a series of decisions to limit potential emotional distress. Our questions are asked in a non-intrusive way. Instead of asking directly about lynching, we present respondents with a typical incident of a thief being punished by neighbors. The term lynching is therefore avoided and other forms of extreme violence (burning, hanging etc.) are not mentioned. Also, participants receive contact details for free psychological counseling in case they feel uneasy after responding to our questions (see consent sheet below).

Legal risks

In our survey, we inquire about experiences with and participation in events similar to lynching. Identifying the drivers of lynching violence is one of the key contributions of our project. However, questions about experiences and participation are sensitive. In particular, the Mexican penal code requires citizens who have first-hand evidence of crimes to report them. This legal obligation in Mexico does not affect our work as we only receive information about crimes via third-person referral.

We understand the tension between, on the one side, the moral obligation to report crimes when becoming aware of them and, on the other side, safeguarding the privacy of study participants. However, if this tension (reporting crimes vs. privacy) was always solved in favor of the moral obligation to report crimes, many of the standard practices in studying violence would be impossible, including the very common practice of qualitative interviews in conflict contexts and victimization surveys, which are used around the globe. Given that a core goal of science is to contribute to the understanding and solution of important societal problems (such as violence), we argue that solving

the tension in favor of protecting the privacy of study participants should be possible under appropriate conditions, exactly to allow for research on the drivers and consequences of violence.

However, this has to be done responsibly, as described for example in the Manual on Victimization Surveys developed by the United Nations Office on Drugs and Crime (UNODC 2010). We devised a series of strategies to mitigate potential concerns. We developed these strategies in collaboration with a Mexican lawyer (UNAM) who has experience in public opinion surveys. In accordance with UNODC guidelines and local guidance from our Mexican partners, our strategy in collecting information about the experience and commission of violent acts is designed to avoid receiving any specific information on concrete crimes.

We do this by referring to hypothetical scenarios and by asking general questions. With the information we receive, it would not be possible to support the prosecution of crimes, unless we asked for additional information from our study participants, which is not in our interest. The information we do receive, does in no case determine a specific act of violence that could be reported to police or judicial institutions. In this sense, we avoid the tension between a moral obligation for reporting crimes and safeguarding the privacy of respondents, as we are unable to report a concrete crime to authorities.

Specific approach taken with regard to the survey question “Have you ever participated in such an event? Yes, No, no answer”: This question refers to a before-mentioned vignette: “A thief assaults a lady on the street. Using a knife, he takes her belongings and escapes. After the robbery, a passer-by manages to take away the thief’s knife and subdues the thief. In this moment, a large number of people gather, insult and punish the thief.”

We understand the sensitivity of our question, which is why we have thought at length about how to formulate it. Before describing our strategy, the key here is that research on self-justice has exclusively focused on support for such violence but not participation in it. We believe that support and participation cannot be equalized and that empirical research on this important societal problem would be largely improved if we had a more direct measure of participation.

Therefore, we developed a strategy (together with our Mexican legal counsellor) to ask this question in a way that would not create a legal or moral obligation to contribute to prosecution. First, our participation question does not refer to an actual crime but to a hypothetical scenario. An affirmative answer – meaning that the surveyee participated in a similar event – does not make it possible for us to contribute to the prosecution of a crime, as we are not informed about any actual crime. We would have to ask additional questions to receive information on an actual crime which could be reported to authorities.

Second, the described scenario does not necessarily describe a crime as the outcome remains legally indeteminate. Citizens are described to insult and punish the thief, terms that have no clear legal implication.

Third, we do not specify the kind of participation. Hence, we do not know whether an affirmative answer to the participation question in fact implies involvement of the surveyee in an actual crime or whether it rather describes active bystanding.

To sum up, given this framing of the question, it would be impossible for us to contribute to the prosecution of a crime.

In addition to the previous, no identifying information about the person who witnessed the hypothetical event or who participated in it is shared with us. Hence, we would not be able to report the participation of a given individual. However, securing anonymity merely provides an additional layer of protection in our study set-up and has to be seen as part of a broader strategy to make it “impossible to draw any conclusions from the answers regarding the commitment of such crimes”, as demanded by the Ethics Board.

Also, in line with the recommendation of our legal adviser we include a note in the consent sheet and offer the possibility of psychological counseling (as mentioned above).

Safety risks for researchers and study participants

When studying violence, researchers may be exposed to risks emanating from their research sites. We devise a series of strategies to minimize risks for the researchers in accordance with guidelines from the safety unit of our university.

With regard to the surveys, we have long debated the mode of survey application. While phone and online survey modes have certain advantages when asking sensitive questions and are less costly,

face-to-face surveying has clear advantages in terms of sampling and data quality. While the pilot survey is conducted online, we decided that the main survey is conducted face-to-face. This decision is the result of an extended conversation with our survey partners of Data OPM. Given our questionnaire and sampling procedures, they estimate the risks for their enumerators to be minimal (in line with most other surveys they have done in the past). They also noted that phone survey participants have become less responsive during the Corona pandemic which makes phone surveys in Mexico extremely burdensome.

The face-to-face survey has safety implications, especially for the enumerators. Our survey partner Data OPM has elaborated a plan to ensure safety (Protocolo de Seguridad). The main points are that during the sampling procedure, locations that are known to be highly insecure are replaced. Further, when enumerators encounter safety problems when arriving at a new location, they abandon this location right away. This is not ideal from the perspective of random sampling, but we obviously weigh the safety of enumerators higher than minor methodological concerns.

Risks from the Corona pandemic

The special situation of the Corona pandemic affords additional safety measures. Researchers adhere to the guidelines of local authorities in Mexico and only travel to field sites if the health risks of their study participants and themselves are manageable.

Our partner Data OPM has their own additional guidelines to reduce the risks to their enumerators ("Protocolo Sanitario por Contingencia COVID"). This includes that no "high-risk" individuals are part of the enumerator team. Enumerators measure their temperature each day before starting with the interviews, disinfect the screens of their mobile devices regularly, use face masks, maintain their distance from each other and their study participants, do not shake hands with participants, do not enter the participants' household, and use private transportation rather than public transport.

More generally speaking, the survey and field visits start no earlier than November 2021 as Mexico is on track to fully vaccinate their adult population by then.

Data protection

Data protection is particularly important in the context of our study, given that we access sensitive information. In principle, all information is anonymized before storage, safely stored on password protected computers, and is only accessible by the authors for the course of the study duration.

All data of the pilot online surveys (conducted with Qualtrics) are fully anonymized, hence, it is not possible for us to attribute any of the responses to specific individuals once we receive the data from Qualtrics. This corresponds to the standard practice by Qualtrics which fully complies with EU's General Data Protection Regulation (GDPR).[8]

Data of the face-to-face survey (conducted by Data OPM) are anonymized. Our survey partner follows the guidelines of the Mexican Federal Law of Personal Data Protection and for this particular project also complies with EU's GDPR. Further, they comply in their standard operating procedures with the ethical code of conduct and best practices of the most important Public Opinion Research Organizations (including the World Association for Public Opinion Research and the European Society for Opinion and Market Research). In case Data OPM receives information that allows for the identification of specific interviewees, for example the exact location of residence, this information will (a) not be shared with the client, and (b) stored separately from the dataset that contains the survey responses. Specifically, Data OPM creates an anonymized key for each respondent to which only their authorized employees have access. Secure file transfer services are used to transfer only the anonymized data at the end of the survey, while the full information on respondents is stored and encrypted on Data OPM's own server and erased after maximally 6 months.

Fully anonymized data of the survey (including questionnaires, codebooks and replication code) will be made publicly available once the respective studies are published, as is standard practice in social science and following FAIR principles for scientific data management (Wilkinson et al. 2016). This is also in line with the spirit of our university's initiative on research data management. Publication of data facilitates replication of our analysis and thus prevents inappropriate data usage.

---

[8] See https://www.qualtrics.com/uk/platform/gdpr/

A5. Additional statistical analyses

Note on missing values

For most analyses, numbers of observations are below the total number of 2183. This is explained by non-response (see summary statistics and regression tables). Survey participants were never forced to give a response. In a survey on issues related to violence, this would be unethical.

Note on imputation

For the creation of multi-item indices, I imputed values for a series of variables. The following variables include imputed values.

- Support for lynching index: All missing values received the arithmetic average between 1 and 4 (i.e. 2.5). The items of this index have between 91 and 31 missing values. Results are very similar without imputing values.
- Individualizing morality, Binding morality, Group-oriented moral beliefs, Harm avoidance foundation, Fairness foundation, Authority foundation, Loyalty foundation, Purity foundation: Before creating these indices, I gave all missing values of individual items measured on a 0-5 scale a value of 3, which is close to the average value for most items. The maximum amount of missing values of a single item is 38. Results are very similar without imputing values.
- Number of light bulbs (an indicator of wealth): All missing values received the arithmetic average between 1 and 4 (i.e. 2.5). This item has 58 missing values. Results are very similar without imputing values.

Single items were always analyzed without imputation, which explains slightly changing numbers of observations across models.

Note on demanding model

Additional control variables for the demanding model include:

- Religious faith: catholic and non-religious, evangelical and other religions are reference categories (Cousar, Carnes, and Kimel 2021).
- Employment status: working and unemployed, retired, student and housekeeping are reference categories.
- Participation in a fight after turning 18 years old to account for individual propensity to use violence.
- Trust in government (Nivette 2016; Levi 1997): additive index including respect for institutions, trust in Armed Forces, trust in police, and trust in justice system. Cronbach alpha: 0.77.
- Adjusting measurement: A variable to correct for systematic bias in the responses to the moral foundations questionnaire. The MFQ contains a question on whether mathematics skills are morally important. This question is not related to the moral domain. If respondents give a high score to this question, they reveal their inattentiveness or misunderstanding of the questions, which may also affect the rest of the questionnaire. I use this variable to adjust for this source of measurement bias.
- Commercial use of the street block (coded by enumerators).
- Public space cleanliness (Vilalta et al. 2020) (coded by enumerators).

A5.1. Summary statistics

	count	mean	sd	min	max
Support for lynching index	2183	1.08	0.98	-1.00	3.50
Agree with lynching	2152	2.94	0.87	1.00	4.00
Would stay and observe lynching	2129	2.16	1.09	1.00	4.00
Would participate in lynching	2120	2.28	1.20	1.00	4.00
Neighbors would support lynching	2092	2.20	1.12	1.00	4.00
Would call the police to denounce	2138	2.58	1.21	1.00	4.00
Perpetrators should be sentenced	2150	2.72	1.19	1.00	4.00
Did stay and observe lynching	2182	0.23	0.42	0.00	1.00
Did participate in lynching	2182	0.10	0.29	0.00	1.00
Suffering for neighbors	2183	0.11	0.32	0.00	1.00
Condemning of neighbors	2183	0.24	0.43	0.00	1.00

Suffering for thief	2183	0.17	0.38	0.00	1.00
Condemning of thief	2183	0.43	0.49	0.00	1.00
Moral prime	2066	0.51	0.50	0.00	1.00
Individualizing morality	2183	3.95	0.78	0.50	5.00
Binding morality	2183	3.54	0.78	0.28	5.00
Group-oriented morals	2183	-0.41	0.65	-2.94	1.89
Harm avoidance foundation	2183	4.03	0.87	0.00	5.00
Fairness foundation	2183	3.87	0.84	0.00	5.00
Authority foundation	2183	3.54	0.90	0.00	5.00
Loyalty foundation	2183	3.69	0.84	0.33	5.00
Purity foundation	2183	3.40	0.97	0.00	5.00
Log of names known in colonia	2159	2.95	1.14	0.00	6.69
Community participation	2169	1.65	0.87	1.00	4.00
Participation in religious activity	2179	2.09	1.19	1.00	4.00
Protest participation	2183	0.05	0.22	0.00	1.00
Torturing suspected terrorists	2168	1.38	1.69	0.00	5.00
Death penalty	2162	3.42	1.86	0.00	5.00
Use of force against immigrants	2169	2.58	1.83	0.00	5.00
Taking up arms to remove corrupt mayor	2174	2.76	1.95	0.00	5.00
Suicide	2146	1.35	1.69	0.00	5.00
Parents allowed to beat children	2177	1.28	1.59	0.00	5.00
Husband allowed to beat wife	2174	0.57	1.24	0.00	5.00
Education	2177	6.61	2.88	0.00	12.00
Age	2176	41.31	15.43	18.00	90.00
Female	2183	0.51	0.50	0.00	1.00
Light bulbs	2183	8.27	6.77	0.00	100.00
Catholic	2183	0.63	0.48	0.00	1.00
Nonreligious	2183	0.21	0.40	0.00	1.00
Working	2183	0.61	0.49	0.00	1.00
Unemployed	2183	0.06	0.24	0.00	1.00
Garbage on street	2183	2.28	0.84	1.00	4.00
Commerce on street	2183	0.63	0.48	0.00	1.00
Fight	2182	0.26	0.44	0.00	1.00
Trust in government	2142	3.91	1.49	1.00	7.00
Adjusting measurement	2168	4.00	1.43	0.00	5.00
N	2183				

A5.2. Summary statistics of colonia-level indicators

	count	mean	sd	min	max
Respondents know of lynching in colonia	340	0.31	0.23	0.00	1.00
Survey-reported number of lynchings in colonia	340	1.60	2.58	0.00	26.67
Newspaper-based lynching events in colonia	340	0.28	0.77	0.00	8.00
Group-oriented morals	340	-0.41	0.31	-1.51	0.54
Population size	340	9085.71	5494.80	474.00	29902.00
Population density	340	17804.18	11025.79	653.46	73135.25
Area in square kilometers	340	0.95	1.59	0.01	11.35
Number of homicides 2009-2020	340	9.81	9.89	0.00	64.00
Garbage on street	340	2.29	0.59	1.00	3.83
N	340				

A5.3. Covariate balance for moral priming

	0	1	(0) vs. (1), p-value
Log of names known in colonia	2.958	2.960	0.961
Community participation	1.710	1.617	0.015
Participation in religious activity	2.081	2.114	0.523
Protest participation	0.058	0.042	0.091
Education	6.562	6.572	0.942

Age	41.744	40.824	0.176
Female	0.516	0.512	0.869
Light bulbs	8.060	8.514	0.131
Catholic	0.630	0.630	0.999
Nonreligious	0.212	0.206	0.734
Working	0.628	0.584	0.040
Unemployed	0.064	0.065	0.891
Garbage on street	2.292	2.290	0.944
Commerce on street	0.626	0.625	0.936
Fight	0.276	0.250	0.174
Trust in government	3.879	3.965	0.191
N	1006	1060	

A5.4. Balance of morality questions depending on moral priming

Note: Answers to the questions regarding morality are balanced. We can thus discard that the answers to these questions depend on whether the lynching questions are displayed before or after them.

	0	1	(0) vs. (1), p-value
Individualizing morality	3.953	3.970	0.622
Binding morality	3.559	3.569	0.774
Group-oriented morals	-0.394	-0.401	0.804
Harm avoidance foundation	4.026	4.054	0.470
Fairness foundation	3.880	3.886	0.873
Authority foundation	3.543	3.587	0.253
Loyalty foundation	3.723	3.697	0.466
Purity foundation	3.411	3.423	0.778
Torturing suspected terrorists	1.473	1.344	0.086
Death penalty	3.398	3.454	0.495
Use of force against immigrants	2.526	2.598	0.368
Taking up arms to remove corrupt mayor	2.822	2.716	0.220
Suicide	1.398	1.324	0.326
Parents allowed to beat children	1.289	1.282	0.912
Husband allowed to beat wife	0.630	0.547	0.131
N	1006	1060	

A5.5. Correlation matrix of lynching items (lynching support and lynching behavior)

	Support for lynching index	Agree with lynching	Would stay and observe lynching	Would participate in lynching	Neighbors would support lynching	Would call the police to denounce	Perpetrators should be sentenced	Did stay and observe lynching	Did participate in lynching
Support for lynching index	1.00								
Agree with lynching	0.62^{***}	1.00							
Would stay and observe lynching	0.56^{***}	0.23^{***}	1.00						
Would participate in lynching	0.71^{***}	0.48^{***}	0.37^{***}	1.00					
Neighbors would support lynching	0.52^{***}	0.18^{***}	0.22^{***}	0.34^{***}	1.00				
Would call	-0.58^{***}	-0.24^{***}	-0.09^{***}	-0.18^{***}	-0.07^{**}	1.00			

the police to denounce									
Perpetrators should be sentenced	-0.57***	-0.23***	-0.09***	-0.17***	-0.04	0.43***	1.00		
Did stay and observe lynching	0.26***	0.13***	0.35***	0.24***	0.21***	-0.01	0.01	1.00	
Did participate in lynching	0.23***	0.15***	0.13***	0.25***	0.22***	-0.05*	-0.03	0.32***	1.00
Observations	1981								

t statistics in parentheses
* $p < 0.05$, ** $p < 0.01$, *** $p < 0.001$

A5.6. The effect of moral priming on lynching support: full regression output for simple model

	(1) Support for lynching index	(2) Agree with lynching	(3) Would stay and observe lynching	(4) Would participate in lynching	(5) Neighbors would support lynching	(6) Would call police to denounce	(7) Perpetrators should be sentenced
Moral prime	-0.26***	-0.18***	-0.14**	-0.27***	-0.12*	0.18**	0.18***
	(0.04)	(0.04)	(0.05)	(0.05)	(0.05)	(0.05)	(0.05)
Education	-0.02**	-0.03***	-0.04***	-0.06***	-0.02	-0.02	-0.02*
	(0.01)	(0.01)	(0.01)	(0.01)	(0.01)	(0.01)	(0.01)
Age	-0.01***	-0.00*	-0.00	-0.01**	-0.01**	0.01***	0.01***
	(0.00)	(0.00)	(0.00)	(0.00)	(0.00)	(0.00)	(0.00)
Female	-0.34***	-0.14***	-0.41***	-0.47***	-0.37***	0.10	-0.06
	(0.04)	(0.04)	(0.05)	(0.05)	(0.05)	(0.05)	(0.05)
Light bulbs	0.00	-0.00	-0.00	0.00	0.01	-0.00	-0.01*
	(0.00)	(0.00)	(0.00)	(0.00)	(0.00)	(0.00)	(0.00)
Constant	1.81***	3.43***	2.76***	3.26***	2.73***	2.30***	2.60***
	(0.10)	(0.09)	(0.11)	(0.12)	(0.11)	(0.12)	(0.12)
N	2054	2028	2004	1998	1981	2018	2028
adj. R^2	0.06	0.02	0.04	0.06	0.03	0.02	0.02

Standard errors in parentheses
OLS models without colonia fixed effects nor clustered standard errors at colonia level.
* $p < 0.05$, ** $p < 0.01$, *** $p < 0.001$

A5.7. The effect of moral priming on lynching support: full regression output for demanding model

	(1) Support for lynching index	(2) Agree with lynching	(3) Would stay and observe lynching	(4) Would participate in lynching	(5) Neighbors would support lynching	(6) Would call police to denounce	(7) Perpetrators should be sentenced
Moral prime	-0.25***	-0.16***	-0.15**	-0.25***	-0.11	0.15*	0.18**
	(0.05)	(0.04)	(0.06)	(0.06)	(0.06)	(0.07)	(0.06)
Education	-0.01	-0.02*	-0.03*	-0.05***	-0.01	-0.02	-0.03*
	(0.01)	(0.01)	(0.01)	(0.01)	(0.01)	(0.01)	(0.01)
Age	-0.01***	-0.00*	-0.00	-0.01*	-0.00*	0.01*	0.01***
	(0.00)	(0.00)	(0.00)	(0.00)	(0.00)	(0.00)	(0.00)
Female	-0.23***	-0.09	-0.33***	-0.35***	-0.24***	0.03	-0.05
	(0.05)	(0.05)	(0.06)	(0.07)	(0.07)	(0.07)	(0.06)
Light bulbs	0.00	-0.00	-0.00	0.00	0.01	-0.00	-0.01*
	(0.01)	(0.00)	(0.01)	(0.01)	(0.01)	(0.01)	(0.00)
Catholic	0.12	0.07	0.06	0.21*	-0.06	-0.08	-0.11
	(0.07)	(0.07)	(0.08)	(0.09)	(0.10)	(0.08)	(0.09)
Nonreligious	0.05	0.07	-0.00	0.02	-0.18	-0.21	-0.06
	(0.09)	(0.08)	(0.10)	(0.10)	(0.11)	(0.11)	(0.10)
Working	-0.03	-0.04	-0.01	0.02	0.08	0.11	0.08
	(0.06)	(0.06)	(0.07)	(0.08)	(0.07)	(0.07)	(0.08)
Unemployed	0.02	0.05	-0.03	0.02	0.05	0.05	-0.01
	(0.11)	(0.10)	(0.13)	(0.14)	(0.13)	(0.15)	(0.14)

Garbage on street	0.02	-0.03	0.01	0.04	0.00	-0.05	-0.04
	(0.04)	(0.04)	(0.04)	(0.05)	(0.05)	(0.05)	(0.05)
Commerce on street	0.11	0.06	-0.01	0.09	0.04	-0.13	-0.13
	(0.06)	(0.06)	(0.07)	(0.08)	(0.07)	(0.08)	(0.07)
Fight	0.31***	0.09	0.18*	0.34***	0.33***	-0.27***	-0.05
	(0.06)	(0.06)	(0.08)	(0.08)	(0.07)	(0.08)	(0.08)
Trust in government	-0.09***	-0.07***	-0.03	-0.06*	-0.04*	0.09***	0.08***
	(0.02)	(0.02)	(0.02)	(0.02)	(0.02)	(0.02)	(0.02)
Adjusting measurement	0.03	0.02	0.04	0.02	0.03	0.01	-0.01
	(0.02)	(0.02)	(0.02)	(0.02)	(0.02)	(0.02)	(0.02)
Constant	1.43***	3.42***	2.67***	2.82***	2.36***	2.81***	2.76***
	(0.20)	(0.19)	(0.23)	(0.24)	(0.25)	(0.27)	(0.27)
N	2003	1980	1956	1952	1936	1970	1981
adj. R^2	0.13	0.05	0.07	0.12	0.07	0.06	0.06

Standard errors in parentheses
OLS models with colonia fixed effects and clustered standard errors at colonia level.
* $p < 0.05$, ** $p < 0.01$, *** $p < 0.001$

A5.8. The effect of moral priming on moral emotions: full regression output for simple models

	(1) Suffering for thief	(2) Condemning of thief
Moral prime	0.07***	-0.06**
	(0.02)	(0.02)
Education	0.01*	0.01**
	(0.00)	(0.00)
Age	0.00	0.00
	(0.00)	(0.00)
Female	0.04*	-0.02
	(0.02)	(0.02)
Light bulbs	0.00	0.00
	(0.00)	(0.00)
Constant	0.03	0.36***
	(0.04)	(0.05)
N	2054	2054
adj. R^2	0.01	0.01

Standard errors in parentheses
OLS models without colonia fixed effects nor clustered standard errors at colonia level. N varies due to non-response.
* $p < 0.05$, ** $p < 0.01$, *** $p < 0.001$

A5.9. The effect of moral priming on moral emotions: full regression output for demanding models

	(1) Suffering for thief	(2) Condemning of thief
Moral prime	0.10***	-0.06*
	(0.02)	(0.03)
Education	0.01**	0.01*
	(0.00)	(0.01)
Age	0.00	0.00
	(0.00)	(0.00)
Female	0.07**	-0.00
	(0.02)	(0.03)
Light bulbs	0.00	0.00
	(0.00)	(0.00)
Catholic	0.02	0.02
	(0.03)	(0.04)
Nonreligious	0.03	0.04
	(0.03)	(0.05)
Working	0.06**	-0.02
	(0.02)	(0.03)
Unemployed	0.04	-0.08

	(0.04)	(0.06)
Garbage on street	0.01	-0.04
	(0.02)	(0.02)
Commerce on street	0.03	-0.03
	(0.03)	(0.03)
Fight	0.06*	0.07*
	(0.03)	(0.03)
Trust in government	0.01	-0.01
	(0.01)	(0.01)
Adjusting measurement	0.01	-0.02*
	(0.01)	(0.01)
Constant	-0.06	0.55***
	(0.09)	(0.11)
N	2003	2003
adj. R^2	0.05	0.03

Standard errors in parentheses
OLS models with colonia fixed effects and clustered standard errors at colonia level. N varies due to non-response.
* $p < 0.05$, ** $p < 0.01$, *** $p < 0.001$

A5.10. The effect of moral priming on lynching participation

The moral priming should not be related to past participation in lynching. Given that we do not see a difference depending on the moral priming, I discard that the effects on lynching support are solely due to social desirability.

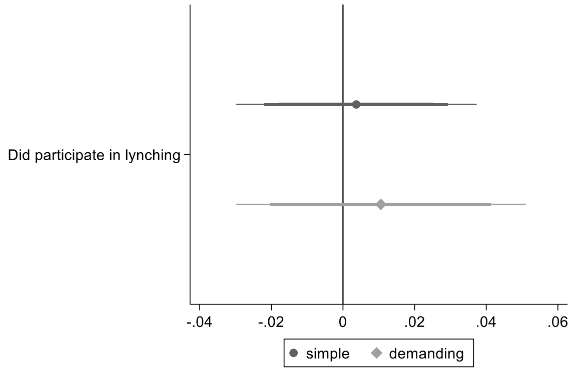

Note: OLS regression coefficients with 99, 95 and 90% confidence intervals. Simple model includes limited number of control variables. Demanding model with additional control variables, clustered standard errors and colonia fixed effects.

A5.11. The effect of moral priming on morality

Moral priming should not be related to morality questions. This would imply that the ordering (lynching questions before morality questions) influences the respondents' moral beliefs. We can discard this option. The index for group-oriented morals is balanced.

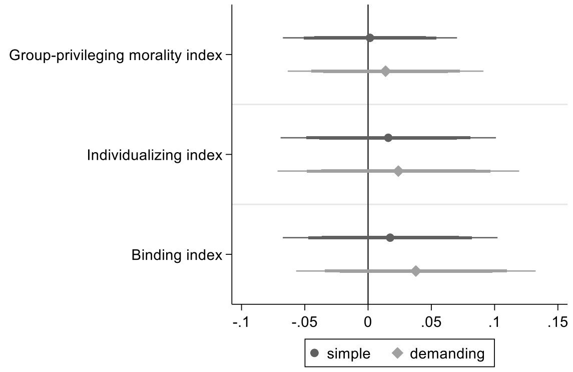

Note: OLS regression coefficients with 99, 95 and 90% confidence intervals. Simple model includes limited number of control variables. Demanding model with additional control variables, clustered standard errors and colonia fixed effects.

A5.12. The association between moral emotions for the target of violence and lynching support

In this figure, we see that moral emotions for the perpetrator are associated with support for lynching. The more emotions of compassion, the less support for lynching (and more support for rejection of lynching – last two items). The more condemning emotions like anger, the more support for lynching. The moral emotion of compassion can thus be interpreted as the emotional dimension of moral judgment.

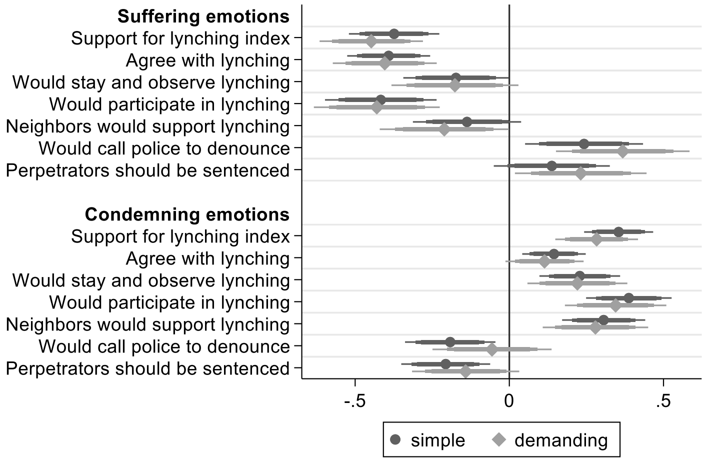

Note: OLS regression coefficients with 99, 95 and 90% confidence intervals. Simple model includes limited number of control variables. Demanding model with additional control variables, clustered standard errors and colonia fixed effects.

A5.13. Group-oriented morals and lynching support: full regression output for simple model

	(1)<br>Support for lynching index	(2)<br>Agree with lynching	(3)<br>Would stay and observe lynching	(4)<br>Would participate in lynching	(5)<br>Neighbors would support lynching	(6)<br>Would call police to denounce	(7)<br>Perpetrators should be sentenced
Group-oriented morals	0.01	0.04	0.07	0.24***	0.04	0.13**	0.20***
	(0.03)	(0.03)	(0.04)	(0.04)	(0.04)	(0.04)	(0.04)
Education	-0.02**	-0.02**	-0.03**	-0.04***	-0.01	-0.01	-0.01
	(0.01)	(0.01)	(0.01)	(0.01)	(0.01)	(0.01)	(0.01)
Age	-0.01***	-0.00*	-0.00	-0.01***	-0.01***	0.01***	0.01**
	(0.00)	(0.00)	(0.00)	(0.00)	(0.00)	(0.00)	(0.00)
Female	-0.36***	-0.15***	-0.40***	-0.46***	-0.36***	0.14*	-0.02
	(0.04)	(0.04)	(0.05)	(0.05)	(0.05)	(0.05)	(0.05)
Light bulbs	0.00	-0.00	-0.00	0.00	0.01	-0.00	-0.01
	(0.00)	(0.00)	(0.00)	(0.00)	(0.00)	(0.00)	(0.00)
Constant	1.68***	3.34***	2.68***	3.16***	2.67***	2.39***	2.72***
	(0.09)	(0.08)	(0.10)	(0.11)	(0.11)	(0.12)	(0.11)
N	2170	2139	2117	2108	2082	2126	2138
adj. R^2	0.05	0.01	0.04	0.07	0.03	0.02	0.03

Standard errors in parentheses

OLS models without colonia fixed effects nor clustered standard errors at colonia level. N varies due to non-response.

* $p < 0.05$, ** $p < 0.01$, *** $p < 0.001$

A5.14. Group-oriented morals and lynching support: full regression output for demanding model

	(1)<br>Support for lynching index	(2)<br>Agree with lynching	(3)<br>Would stay and observe lynching	(4)<br>Would participate in lynching	(5)<br>Neighbors would support lynching	(6)<br>Would call police to denounce	(7)<br>Perpetrators should be sentenced
Group-oriented	0.09*	0.10*	0.11*	0.27***	0.06	0.03	0.15**
	(0.04)	(0.04)	(0.05)	(0.05)	(0.05)	(0.05)	(0.05)

morals							
Education	-0.01	-0.02	-0.02	-0.03*	-0.01	-0.02	-0.03
	(0.01)	(0.01)	(0.01)	(0.01)	(0.01)	(0.01)	(0.01)
Age	-0.01***	-0.00*	-0.00	-0.01***	-0.01**	0.01**	0.01**
	(0.00)	(0.00)	(0.00)	(0.00)	(0.00)	(0.00)	(0.00)
Female	-0.23***	-0.08	-0.32***	-0.32***	-0.25***	0.04	-0.03
	(0.05)	(0.05)	(0.06)	(0.06)	(0.07)	(0.07)	(0.06)
Light bulbs	0.00	-0.00	0.00	0.00	0.01	-0.00	-0.01*
	(0.00)	(0.00)	(0.00)	(0.00)	(0.01)	(0.01)	(0.00)
Catholic	0.11	0.06	0.07	0.16	-0.04	-0.08	-0.11
	(0.07)	(0.06)	(0.08)	(0.09)	(0.09)	(0.08)	(0.08)
Nonreligious	0.06	0.09	0.04	0.03	-0.15	-0.18	-0.01
	(0.09)	(0.08)	(0.09)	(0.10)	(0.10)	(0.10)	(0.10)
Working	-0.03	-0.05	-0.03	0.02	0.06	0.08	0.06
	(0.06)	(0.06)	(0.06)	(0.07)	(0.07)	(0.07)	(0.07)
Unemployed	0.03	0.06	-0.02	0.03	0.04	0.04	-0.03
	(0.11)	(0.10)	(0.13)	(0.13)	(0.13)	(0.14)	(0.14)
Garbage on	0.02	-0.03	0.02	0.02	0.00	-0.04	-0.04
street	(0.04)	(0.03)	(0.04)	(0.04)	(0.04)	(0.05)	(0.04)
Commerce on	0.11	0.08	-0.01	0.09	0.03	-0.11	-0.14
street	(0.06)	(0.06)	(0.07)	(0.08)	(0.07)	(0.08)	(0.07)
Fight	0.31***	0.10	0.17*	0.35***	0.31***	-0.27***	-0.06
	(0.06)	(0.06)	(0.08)	(0.08)	(0.07)	(0.08)	(0.08)
Trust in	-0.11***	-0.08***	-0.04*	-0.08***	-0.06**	0.10***	0.08***
government	(0.02)	(0.02)	(0.02)	(0.02)	(0.02)	(0.02)	(0.02)
Adjusting	0.02	0.01	0.04	0.01	0.03	0.01	-0.03
measurement	(0.02)	(0.02)	(0.02)	(0.02)	(0.02)	(0.02)	(0.02)
Constant	1.41***	3.40***	2.62***	2.86***	2.37***	2.81***	2.84***
	(0.20)	(0.18)	(0.22)	(0.24)	(0.23)	(0.26)	(0.25)
N	2115	2088	2065	2058	2033	2074	2087
adj. R^2	0.12	0.05	0.07	0.12	0.07	0.07	0.07

Standard errors in parentheses
OLS models with colonia fixed effects and clustered standard errors at colonia level. N varies due to non-response.
* $p < 0.05$, ** $p < 0.01$, *** $p < 0.001$

A5.15. Group-oriented morals and lynching behavior: full regression output for simple model

	(1)	(2)
	Did stay and observe lynching	Did participate in lynching
Group-oriented morals	0.04*	0.02
	(0.01)	(0.01)
Education	-0.00	0.00
	(0.00)	(0.00)
Age	0.00	-0.00
	(0.00)	(0.00)
Female	-0.13***	-0.07***
	(0.02)	(0.01)
Light bulbs	-0.00	0.00
	(0.00)	(0.00)
Constant	0.33***	0.14***
	(0.04)	(0.03)
N	2169	2169
adj. R^2	0.03	0.01

Standard errors in parentheses
OLS models without colonia fixed effects nor clustered standard errors at colonia level.
* $p < 0.05$, ** $p < 0.01$, *** $p < 0.001$

A5.16. Group-oriented morals and lynching behavior: full regression output for demanding model

	(1)	(2)

	Did stay and observe lynching	Did participate in lynching
Group-oriented morals	0.03	0.02
	(0.02)	(0.01)
Education	0.00	0.00
	(0.00)	(0.00)
Age	0.00	0.00
	(0.00)	(0.00)
Female	-0.06**	-0.02
	(0.02)	(0.02)
Light bulbs	-0.00	0.00
	(0.00)	(0.00)
Catholic	0.01	-0.05*
	(0.03)	(0.02)
Nonreligious	0.00	-0.05
	(0.04)	(0.03)
Working	0.05*	0.01
	(0.02)	(0.02)
Unemployed	0.01	0.02
	(0.05)	(0.03)
Garbage on street	-0.01	-0.01
	(0.02)	(0.01)
Commerce on street	-0.02	-0.01
	(0.03)	(0.02)
Fight	0.15***	0.14***
	(0.03)	(0.02)
Trust in government	-0.02**	-0.01
	(0.01)	(0.01)
Adjusting measurement	0.01	-0.00
	(0.01)	(0.01)
Constant	0.30***	-0.00
	(0.08)	(0.06)
N	2114	2115
adj. R^2	0.08	0.07

Standard errors in parentheses
OLS models with colonia fixed effects and clustered standard errors at colonia level. N varies due to non-response.
* $p < 0.05$, ** $p < 0.01$, *** $p < 0.001$

A5.17. Binding and individualizing morality and support for lynching

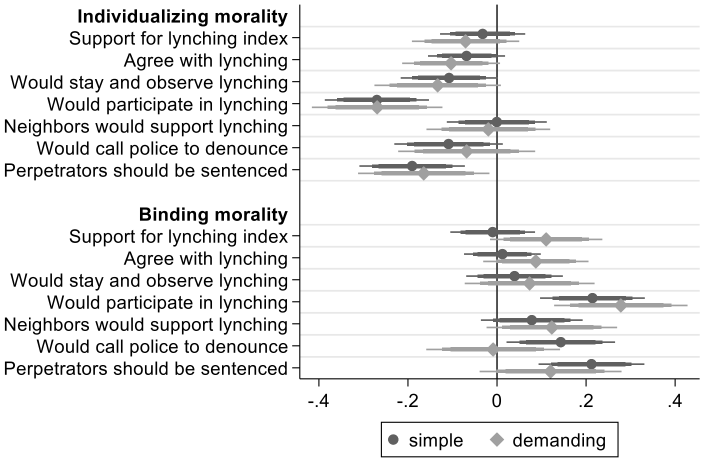

Note: OLS regression coefficients with 99, 95 and 90% confidence intervals. Simple model includes limited number of control variables. Demanding model with additional control variables, clustered standard errors and colonia fixed effects.

Note: The relationship with items indicating rejection of lynching (calling the police and sentencing perpetrators) is most ambiguous, perhaps because of the fraught relationship between citizens and the criminal justice system often seen as corrupt and inefficient (Azaola Garrido 2006).

A5.18. Binding and individualizing morality and lynching behavior

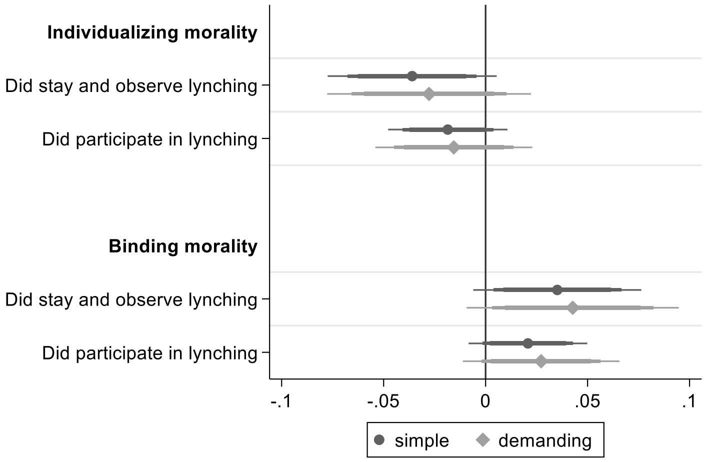

Note: OLS regression coefficients with 99, 95 and 90% confidence intervals. Simple model includes limited number of control variables. Demanding model with additional control variables, clustered standard errors and colonia fixed effects.

A5.19. Group-oriented morals and other forms of participation

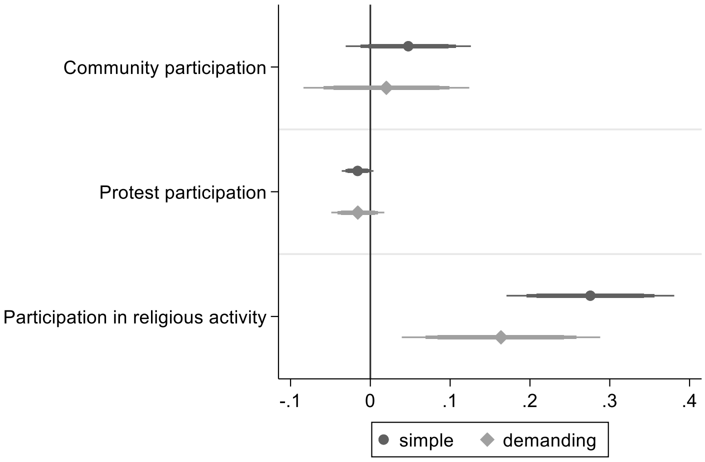

Note: OLS regression coefficients with 99, 95 and 90% confidence intervals. Simple model includes limited number of control variables. Demanding model with additional control variables, clustered standard errors and colonia fixed effects.

A5.20. Group-based moral beliefs and lynching on the colonia level (N=340)

For this aggregate analysis, I created a colonia-level dataset of Mexico City including a series of social and geographic characteristics based on the coding of geo-located census and other administrative information (see description above: A3). I use the mean of group-oriented morals of respondents from the same colonia as colonia-level indicator for group-oriented moral beliefs (ranging from -1.51 to +0.54).[9]

As outcome variables, I use three indicators. First, an aggregate estimate of whether respondents know of lynchings in their colonia or not (31% respond affirmatively). Second, the average number of lynchings reported by respondents in each colonia (in response to the question: "How many lynching-style cases in your colonia have you heard of in the last 5 years"). This variable ranges from 0 to 27 with a mean of 1.6 (I use the natural log). Third, the lynching events recorded in the original newspaper-based dataset aggregated to the colonia level covering 2000 to February 2022. In the 340 colonias, we registered an average of 0.17 lynchings ranging from 0 to 9 (I use the natural log). I again

[9] To estimate colonia-level indicators, we use the 6 originally sampled individuals in each colonia, which were block-randomized to experimental treatments. For each colonia, this provides us with a systematically unbiased sample, but large random bias, due to the small number of observations within each colonia. Findings have suggestive character.

show a simple model (accounting only for colonia-level population size, population density, and area)[10] and a demanding model (additionally including colonia level homicides, public space cleanliness, borough-level fixed effects and borough-level clustered standard errors).

The below figure shows a positive relationship between group-oriented moral beliefs aggregated to the colonia level and all three indicators of lynching. Again, we need to assume that moral beliefs are relatively stable as the dependent variable of this analysis refers to the past.

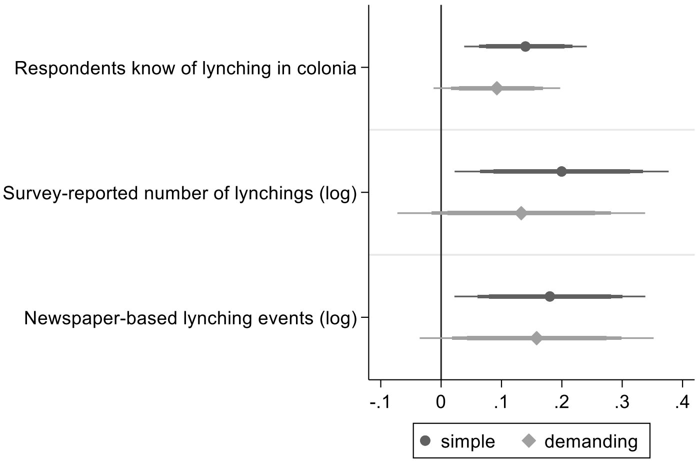

Note: OLS regression coefficients with 99, 95 and 90% confidence intervals. Simple model includes limited number of control variables. Demanding model with additional control variables, clustered standard errors and alcaldía fixed effects.

A5.21. Group-oriented morals and moral emotions: full regression output for simple model

	(1)<br>Suffering for thief	(2)<br>Condemning of thief
Group-oriented morals	-0.06***	-0.01
	(0.01)	(0.02)
Education	0.00	0.01***
	(0.00)	(0.00)
Age	0.00*	0.00
	(0.00)	(0.00)
Female	0.04*	-0.03
	(0.02)	(0.02)
Light bulbs	0.00	0.00
	(0.00)	(0.00)
Constant	0.05	0.33***
	(0.04)	(0.05)
N	2170	2170
adj. R^2	0.01	0.01

Standard errors in parentheses
OLS models without colonia fixed effects nor clustered standard errors at colonia level. N varies due to non-response.
* $p < 0.05$, ** $p < 0.01$, *** $p < 0.001$

A5.22. Group-oriented morals and moral emotions: full regression output for demanding model

	(1)<br>Suffering for thief	(2)<br>Condemning of thief
Group-oriented morals	-0.05**	-0.02
	(0.02)	(0.02)
Education	0.01*	0.01*
	(0.00)	(0.01)
Age	0.00	0.00
	(0.00)	(0.00)

[10] It is important to account for population indicators as colonias were selected using probability proportional to (population) size sampling.

Female	0.07**	-0.01
	(0.02)	(0.03)
Light bulbs	0.00	0.00
	(0.00)	(0.00)
Catholic	0.03	0.03
	(0.03)	(0.04)
Nonreligious	0.03	0.03
	(0.03)	(0.05)
Working	0.06*	-0.02
	(0.02)	(0.03)
Unemployed	0.04	-0.09
	(0.04)	(0.05)
Garbage on street	0.01	-0.02
	(0.02)	(0.02)
Commerce on street	0.02	-0.05
	(0.02)	(0.03)
Fight	0.06*	0.06
	(0.02)	(0.03)
Trust in government	0.01	-0.01
	(0.01)	(0.01)
Adjusting measurement	0.01	-0.01
	(0.01)	(0.01)
Constant	-0.01	0.50***
	(0.08)	(0.10)
N	2115	2115
adj. R^2	0.04	0.03

Standard errors in parentheses
OLS models with colonia fixed effects and clustered standard errors at colonia level. N varies due to non-response.
* $p < 0.05$, ** $p < 0.01$, *** $p < 0.001$

A5.23. Moral emotions for target and lynching behavior

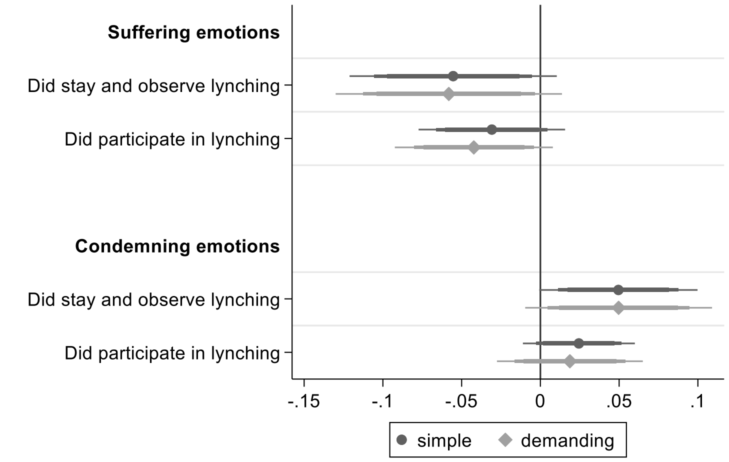

Note: OLS regression coefficients with 99, 95 and 90% confidence intervals. Simple model includes limited number of control variables. Demanding model with additional control variables, clustered standard errors and colonia fixed effects.

A5.24 Sensitivity analysis

To examine the sensitivity of key findings in observational analysis, I follow procedures recommended by Emily Oster (2019). Oster's procedure is based on the following intuition: if relevant coefficients of regression models are stable after including additional covariates that produce large movements in R^2 (overall model fit), the risk of omitted variable bias is limited. For another application of this procedure, see also Tsai et al. (2020).

I examine the sensitivity of two key variables: past participation in lynching and whether an individual would participate in the future. As some of the other variables are not strongly related to group-oriented morals, examining their sensitivity is less meaningful.

To implement this procedure, I need to specify the potential influence of unobserved covariates (delta) and the maximum level of R^2:

- In line with Oster's recommendations, I use a *delta of 1*. This means that unobserved covariates are equally influential as observed covariates. This is a highly penalizing assumption considering that the adjusted models used in the main paper address the most important sources of confounding and include colonia-level fixed effects, thus mitigating the possibility of context-level confounding.
- In line with Oster's recommendation, I use a *Rmax of 1.3*, which is derived from experimental studies in economics.

The below Table presents coefficients adjusted for the specified delta and Rmax (the bias-adjusted coefficient) along with the naïve coefficients (from models without control variables) and controlled coefficients (from models with control variables). I replicate the same regressions as in the main paper focusing on group-oriented moral beliefs and different outcomes in a simple and demanding model specification.

Comparing coefficients from a naïve and a controlled framework, we see little movement in coefficient sizes, despite large increases in R^2 – especially in the demanding models which include the full set of control variables. In accordance with this general intuition, bias adjusted coefficients remain in a similar range as the naïve and controlled coefficients, suggesting limited omitted variable bias.

Table. Sensitivity analysis of coefficients using Oster's procedures

Dependent variable	Model specification	Naïve coefficient (R^2)	Controlled coefficient (R^2)	Bias-adjusted coefficient – Oster's beta
Participation in lynching	Simple	0.021 (0.002)	0.020 (0.015)	0.019
Participation in lynching	Demanding	0.022 (0.002)	0.021 (0.22)	0.020
Would participate	Simple	0.29 (0.025)	0.24 (0.072)	0.22
Would participate	Demanding	0.30 (0.026)	0.27 (0.275)	0.26

A6. Links to questionnaire and consent sheet

Due to the length of the full survey questionnaire, I include a link to the original and translated versions.

Full survey questionnaire (original Spanish):
https://www.dropbox.com/s/fx9xdj2le5s9jnq/Questionnaire%20Espanol%20BLINDED.pdf?dl=0

Full survey questionnaire (English translation):
https://www.dropbox.com/s/1j49hmkne1c5t5q/Questionnaire%20English%20BLINDED.pdf?dl=0

Consent sheet (original Spanish):
https://www.dropbox.com/s/d08z0m51h8bse4y/Consent%20sheet%20Espanol%20BLINDED.pdf?dl=0

Consent sheet (English translation):
https://www.dropbox.com/s/ttk9mc3jel0nh6n/Consent%20sheet%20English%20BLINDED.pdf?dl=0

A7. References

Azaola Garrido, Elena. 2006. *Imagen y autoimagen de la polícia de la Ciudad de México*. Mexico City: Ediciones Coyoacán.

Benítez, Fernando. 1992. *El agua envenenada*. México, D.F: FCE.

CNDH. 2019. "Informe Especial Sobre Los Linchamientos En México." Mexico: Comisión Nacional de Derechos Humanos.

Cohen, Taya R., R. Matthew Montoya, and Chester A. Insko. 2006. "Group Morality and Intergroup Relations: Cross-Cultural and Experimental Evidence." *Personality and Social Psychology Bulletin* 32 (11): 1559–72.

Cousar, Kayleigh A., Nate C. Carnes, and Sasha Y. Kimel. 2021. "Morality as Fuel for Violence? Disentangling the Role of Religion in Violent Conflict." *Social Cognition* 39 (1): 166–82.

García-Sánchez, Miguel, and Rosario Queirolo. 2020. "A Tale of Two Countries. The Functioning of List Experiments to Measure Drug Consumption in Opposite Contexts."

Herrero, Juan, Francisco J. Rodríguez, and Andrea Torres. 2017. "Acceptability of Partner Violence in 51 Societies: The Role of Sexism and Attitudes Toward Violence in Social Relationships." *Violence Against Women* 23 (3): 351–67.

Hornor, Gail, Deborah Bretl, Evelyn Chapman, Ellen Chiocca, Carrie Donnell, Katharine Doughty, Susan Houser, Bridget Marshall, Kristen Morris, and Saribel Garcia Quinones. 2015. "Corporal Punishment: Evaluation of an Intervention by PNPs." *Journal of Pediatric Health Care* 29 (6): 526–35.

Levi, Margaret. 1997. *Consent, Dissent, and Patriotism*. Cambridge: Cambridge University Press.

Mooijman, Marlon, Peter Meindl, Daphna Oyserman, John Monterosso, Morteza Dehghani, John M. Doris, and Jesse Graham. 2018. "Resisting Temptation for the Good of the Group: Binding Moral Values and the Moralization of Self-Control." *Journal of Personality and Social Psychology* 115 (3): 585–99.

Nivette, Amy E. 2016. "Institutional Ineffectiveness, Illegitimacy, and Public Support for Vigilantism in Latin America." *Criminology* 54 (1): 142–75.

Nussio, Enzo. 2023. "Replication Data for: How Moral Beliefs Influence Collective Violence. Evidence from Lynching in Mexico." Harvard Dataverse. https://doi.org/10.7910/DVN/X6E6XC.

Oster, Emily. 2019. "Unobservable Selection and Coefficient Stability: Theory and Evidence." *Journal of Business & Economic Statistics* 37 (2): 187–204.

Skinner, Chris J. 2016. "Probability Proportional to Size (PPS) Sampling." In *Wiley StatsRef*, 1–5. New York: John Wiley & Sons, Ltd.

Slovic, Paul, C. K. Mertz, David M. Markowitz, Andrew Quist, and Daniel Västfjäll. 2020. "Virtuous Violence from the War Room to Death Row." *Proceedings of the National Academy of Sciences* 117 (34): 20474–82.

Smith, Isaac H., Karl Aquino, Spassena Koleva, and Jesse Graham. 2014. "The Moral Ties That Bind . . . Even to Out-Groups: The Interactive Effect of Moral Identity and the Binding Moral Foundations." *Psychological Science* 25 (8): 1554–62.

Sundberg, Ralph. 2014. "Violent Values: Exploring the Relationship between Human Values and Violent Attitudes." *Peace and Conflict: Journal of Peace Psychology* 20 (1): 68–83.

Tsai, Lily L., Benjamin S. Morse, and Robert A. Blair. 2020. "Building Credibility and Cooperation in Low-Trust Settings: Persuasion and Source Accountability in Liberia During the 2014–2015 Ebola Crisis." *Comparative Political Studies* 53 (10–11): 1582–1618.

UNODC. 2010. "Manual on Victimization Surveys." Geneva: UNODC. https://www.unodc.org/documents/data-and-analysis/Crime-statistics/Manual_on_Victimization_surveys_2009_web.pdf.

Vilalta, Carlos J., Pablo Lopez, Gustavo Fondevila, and Oscar Siordia. 2020. "Testing Broken Windows Theory in Mexico City." *Social Science Quarterly* 101 (2): 558–72.

Vilalta, Carlos J., Robert Muggah, and Gustavo Fondevila. 2020. "Homicide as a Function of City Block Layout: Mexico City as Case Study." *Global Crime*, 1–19.

Wilkinson, Mark D., Michel Dumontier, IJsbrand Jan Aalbersberg, Gabrielle Appleton, Myles Axton, Arie Baak, Niklas Blomberg, et al. 2016. "The FAIR Guiding Principles for Scientific Data Management and Stewardship." *Scientific Data* 3 (1): 160018.
